# A holistic genome dataset of bacteria and archaea of mangrove sediments

**DOI:** 10.1093/gigascience/giaf081

**Published:** 2025-08-12

**Authors:** Shijun Pan, Huan Du, Ruiqi Zheng, Cuijing Zhang, Jie Pan, Xilan Yang, Cheng Wang, Xiaolan Lin, Jinhui Li, Wan Liu, Haokui Zhou, Xiaoli Yu, Shuming Mo, Guoqing Zhang, Guoping Zhao, Zhili He, Yun Tian, Chengjian Jiang, Wu Qu, Yang Liu, Meng Li

**Affiliations:** Archaeal Biology Centre, Synthetic Biology Research Center, Shenzhen Key Laboratory of Marine Microbiome Engineering, Key Laboratory of Marine Microbiome Engineering of Guangdong Higher Education Institutes, Institute for Advanced Study, Shenzhen University, Shenzhen 518060, China; Archaeal Biology Centre, Synthetic Biology Research Center, Shenzhen Key Laboratory of Marine Microbiome Engineering, Key Laboratory of Marine Microbiome Engineering of Guangdong Higher Education Institutes, Institute for Advanced Study, Shenzhen University, Shenzhen 518060, China; Shenzhen Key Laboratory of Synthetic Genomics, Guangdong Provincial Key Laboratory of Synthetic Genomics, CAS Key Laboratory of Quantitative Engineering Biology, Shenzhen 518055, China; Institute of Synthetic Biology, Shenzhen Institutes of Advanced Technology, Chinese Academy of Sciences, Shenzhen 518055, China; Archaeal Biology Centre, Synthetic Biology Research Center, Shenzhen Key Laboratory of Marine Microbiome Engineering, Key Laboratory of Marine Microbiome Engineering of Guangdong Higher Education Institutes, Institute for Advanced Study, Shenzhen University, Shenzhen 518060, China; Archaeal Biology Centre, Synthetic Biology Research Center, Shenzhen Key Laboratory of Marine Microbiome Engineering, Key Laboratory of Marine Microbiome Engineering of Guangdong Higher Education Institutes, Institute for Advanced Study, Shenzhen University, Shenzhen 518060, China; Shenzhen Key Laboratory of Synthetic Genomics, Guangdong Provincial Key Laboratory of Synthetic Genomics, CAS Key Laboratory of Quantitative Engineering Biology, Shenzhen 518055, China; Institute of Synthetic Biology, Shenzhen Institutes of Advanced Technology, Chinese Academy of Sciences, Shenzhen 518055, China; Southern Marine Science and Engineering Guangdong Laboratory (Zhuhai), Zhuhai 519080, China; State Key Laboratory for Biocontrol, School of Marine Sciences, School of Environmental Science and Engineering, Sun Yat-sen University, Guangzhou 510006, China; Key Laboratory of the Ministry of Education for Coastal and Wetland Ecosystems, School of Life Sciences, Xiamen University, Xiamen 361102, China; Guangxi Key Laboratory for Green Processing of Sugar Resources, College of Biological and Chemical Engineering, Guangxi University of Science and Technology, Liuzhou 545006, China; National Genomics Data Center & Bio-Med Big Data Center, CAS Key Laboratory of Computational Biology, Shanghai Institute of Nutrition and Health, University of Chinese Academy of Sciences, Chinese Academy of Science, Shanghai 200031, China; Shenzhen Key Laboratory of Synthetic Genomics, Guangdong Provincial Key Laboratory of Synthetic Genomics, CAS Key Laboratory of Quantitative Engineering Biology, Shenzhen 518055, China; Institute of Synthetic Biology, Shenzhen Institutes of Advanced Technology, Chinese Academy of Sciences, Shenzhen 518055, China; Southern Marine Science and Engineering Guangdong Laboratory (Zhuhai), Zhuhai 519080, China; State Key Laboratory for Biocontrol, School of Marine Sciences, School of Environmental Science and Engineering, Sun Yat-sen University, Guangzhou 510006, China; Guangxi Key Laboratory for Green Processing of Sugar Resources, College of Biological and Chemical Engineering, Guangxi University of Science and Technology, Liuzhou 545006, China; National Genomics Data Center & Bio-Med Big Data Center, CAS Key Laboratory of Computational Biology, Shanghai Institute of Nutrition and Health, University of Chinese Academy of Sciences, Chinese Academy of Science, Shanghai 200031, China; National Genomics Data Center & Bio-Med Big Data Center, CAS Key Laboratory of Computational Biology, Shanghai Institute of Nutrition and Health, University of Chinese Academy of Sciences, Chinese Academy of Science, Shanghai 200031, China; Hangzhou Institute for Advanced Study, University of Chinese Academy of Sciences, Hangzhou 310024, China; Southern Marine Science and Engineering Guangdong Laboratory (Zhuhai), Zhuhai 519080, China; State Key Laboratory for Biocontrol, School of Marine Sciences, School of Environmental Science and Engineering, Sun Yat-sen University, Guangzhou 510006, China; Key Laboratory of the Ministry of Education for Coastal and Wetland Ecosystems, School of Life Sciences, Xiamen University, Xiamen 361102, China; Guangxi Key Laboratory for Green Processing of Sugar Resources, College of Biological and Chemical Engineering, Guangxi University of Science and Technology, Liuzhou 545006, China; Marine Science and Technology College, Zhejiang Ocean University, Zhoushan 316022, China; Archaeal Biology Centre, Synthetic Biology Research Center, Shenzhen Key Laboratory of Marine Microbiome Engineering, Key Laboratory of Marine Microbiome Engineering of Guangdong Higher Education Institutes, Institute for Advanced Study, Shenzhen University, Shenzhen 518060, China; Archaeal Biology Centre, Synthetic Biology Research Center, Shenzhen Key Laboratory of Marine Microbiome Engineering, Key Laboratory of Marine Microbiome Engineering of Guangdong Higher Education Institutes, Institute for Advanced Study, Shenzhen University, Shenzhen 518060, China

**Keywords:** microbial composition, mangrove wetland, sediment microbiome, metagenome sequencing, metagenome-assembled genomes

## Abstract

**Background:**

Mangroves are one of the most productive marine ecosystems with high ecosystem service value. The sediment microbial communities contribute to pivotal ecological functions in mangrove ecosystems. However, the study of mangrove sediment microbiomes is limited.

**Findings:**

Here, we applied metagenome sequencing analysis of microbial communities in mangrove sediments across Southeast China from 2014 to 2020. This genome dataset includes 966 metagenome-assembled genomes with ≥50% completeness and ≤10% contamination generated from 6 groups of samples. Phylogenomic analysis and taxonomy classification show that mangrove sediments are inhabited by microbial communities with high species diversity. Thermoplasmatota, Thermoproteota, and Asgardarchaeota in archaea, as well as Proteobacteria, Desulfobacterota, Chloroflexota, Acidobacteriota, and Gemmatimonadota in bacteria, dominate the mangrove sediments across Southeast China. Functional analyses suggest that the microbial communities may contribute to carbon, nitrogen, and sulfur cycling in mangrove sediments.

**Conclusions:**

These combined microbial genomes provide an important complement of global mangrove genome datasets and may serve as a foundational resource for enhancing our understanding of the composition and functions of mangrove sediment microbiomes.

## Background

Mangroves are high-productivity ecosystems growing in swamp tidal areas of tropical and subtropical coastal areas. As an important part of coastal “blue carbon sink,” they play an extremely important role in purifying seawater, preventing waves, maintaining biodiversity, and fixing carbon [1–5]. Mangroves provide a unique ecological environment. The special ecological characteristics, such as the dynamic change of salinity, large organic matter storage, anoxia, and high redox potential value, create a high diversity and abundance of a microbial community [6]. Similar to typical terrestrial plants, mangroves depend on mutually beneficial interaction with microbial communities [7]. The close relationship between microorganism, nutrient, and plant promotes the cycle and preservation of main nutrients (carbon, nitrogen, phosphorus, and sulfur), which is helpful to maintain the high productivity of the mangrove ecosystem and further improve mangrove vegetation [8]. However, the mangrove microbial community is threatened by global climate change and human factors such as coastal development, aquaculture, and logging activities. It is urgent to take appropriate protection measures to deeply understand and protect this unique ecological community [9, 10]. Although there have been many studies on the mangrove ecosystem, the global exploration on microbial composition, distribution, interaction, and ecological function of its sediments is still insufficient. Therefore, the dataset of mangrove sediment microbiomes represents a valuable resource, which could enhance our understanding of the composition, structure, and putative functional roles of mangrove sediment microbiomes.

Mangroves in China are mainly distributed in Fujian, Zhejiang, Guangdong, Guangxi, Hainan, Hong Kong, Macao, and Taiwan along the southeast coast, with a total area of about 28,900 hectares, accounting for 0.2% of the world. Due to the urbanization of coastal areas, the construction of ports and development zones, and the openness, fragility, and complexity of the mangrove ecosystem, its distribution is seriously affected by human activities [11, 12]. For this reason, China has established several mangrove nature reserves, which have alleviated the destruction and disappearance of mangroves to a certain extent, but the mangroves outside the reserves (often broken mangroves) have not improved significantly [13]. Although some ecological and genomic studies have been carried out on the microbial community of mangroves along the southeast coast of China [14–16], a comprehensive dataset of the microbial community, including sediments of mangrove nature reserves, needs to be improved. Acknowledging the existing gaps in our understanding, including limited studies on microbial communities within mangrove sediments and the fragmented nature of mangrove conservation efforts, collaborative initiatives such as the Mangrove Microbiome Initiative (MMI) have been established [17]. Through this international network of researchers, concerted efforts are being made to enhance the comprehensive dataset pertaining to microbial communities, especially within the sediments of mangrove nature reserves. This collaborative approach is crucial for advancing our understanding of microbial diversity, functionality, and evolutionary dynamics within mangrove ecosystems both in China and globally.

**Figure 1:**
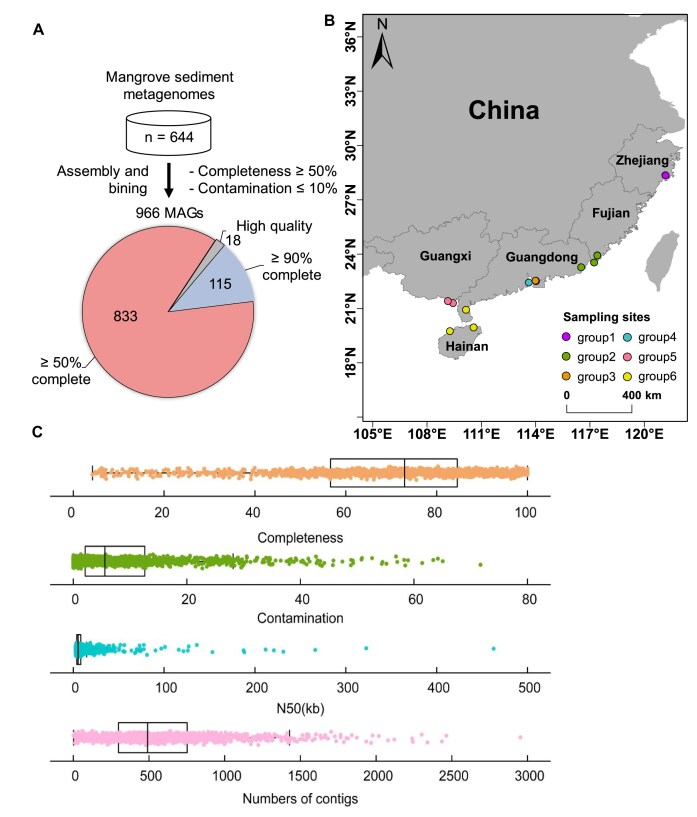
Geographical distribution of metagenome-assembled genomes. (A) A total of 966 MAGs were recovered from the mangrove sediment metagenomes. Most metagenomes were reassembled for this work using the latest state-of-the-art assembly pipeline. These genomes form the MSM catalog. All MAGs were ≥50% complete and ≤5% contaminated. (B) Geographic distribution of the 6 groups of sediment sites where metagenomic sequencing data were collected. (C) Genome statistics for the representative species of nonredundant MAGs, showing the minimum value, first quartile, median, third quartile, and maximum value.

In this study, we deeply sequenced the metagenomes of 6 groups of sediments collected in mangrove wetlands along the southeast coast of China from 2014 to 2020, spanning mangroves in 6 nature reserves (Table 1 and Fig. 1B). We recovered 966 medium- and high-quality metagenome-assembled genomes (MAGs), which form the genomes from the bacteria-focused and archaea-focused mangrove sediment metagenome (MSM) catalog (Fig. 1A). The MSM catalog was constructed from 644 metagenomes from these mangrove natural reserves of China. The nonredundant gene and MSM catalog is a valuable resource that will aid in deepening our understanding of the composition, structure, and functions of mangrove sediment microbiomes.

**Table 1:** Summary of reads, contigs, and MAGs of MSMs

Group	Read pairs after QC	Contigs (>2,000 bp)	Prokaryotic MAGs*
1	589,800,176	572,210	117
2	1,998,091,443	1,423,185	123
3	4,271,704,109	3,743,050	296
4	838,714,126	498,188	60
5	1,392,266,226	447,022	41
6	645,225,166	532,975	93

* Completeness ≥50%, contamination ≤10%.

## Data Description

### Summary of reads, contigs, and MSMs of mangrove sediment metagenomes

We performed metagenomic assembly and binning on 644 metagenomes from 6 mangrove natural reserves in 5 provinces, including Fujian (248), Guangxi (183), Guangdong (120), Zhejiang (65), and Hainan (28) (Fig. 1B). Overall, 7,216,630 contigs (>2,000 bp) were generated by assembling quality-checked sequencing reads (Table 1). This catalog of MAGs contains representatives from all mangrove wetlands along the southeast coast of China. A total of 966 MAGs from the MSM catalog were reconstructed based on multistrategy binning according to the MIMAG criteria [18] (mean completeness = 75.57%; mean contamination = 4.09%) and include 13 assigned as high quality based on the presence of a near-full complement of ribosomal RNAs (rRNAs), transfer RNAs (tRNAs), and single-copy protein-coding genes (Fig. 1A, C). The genome sizes of these MSMs ranged from 0.26 to 7.76 Mb, and GC content varied from 22% to 74%. Most small-sized MAGs belong to Aenigmatarchaeota, Nanoarchaeota, or Patescibacteria, and similarly, large-sized MAGs belong to Acidobacteriota or Desulfobacterota.

### Functional annotation and taxonomic classification of the MSM catalogs

**Figure 2:**
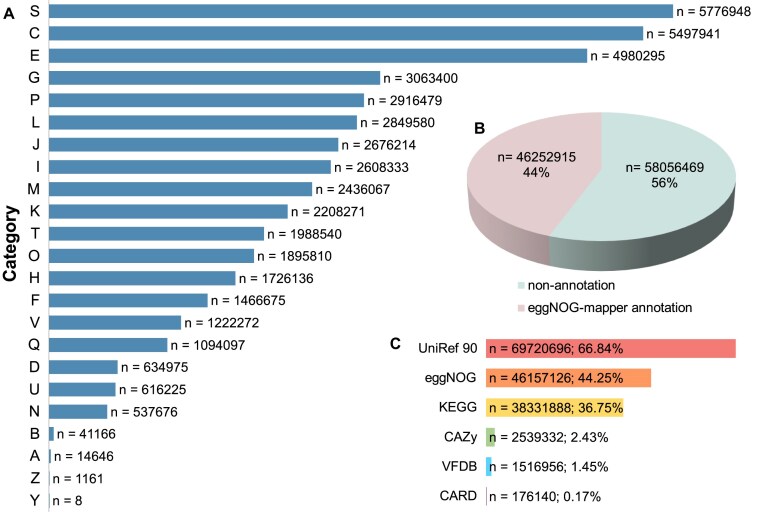
Functional annotation and taxonomic classification of the MSM catalog. (A) Functional annotations at the eggNOG category level. S: Function unknown. (B) An overview of annotations for the nonredundant gene catalog. Nonannotation indicates that these genes were not annotated in at least one of the following databases: eggNOG, KEGG, UniRef 90, VFDB, CARD, and CAZy. (C) Number of genes with functional annotations across the 6 functional databases.

The functional annotations, including those for eggnog 5.0 [19] (evolutionary genealogy of genes: Non-supervised Orthologous), KEGG [20] (Kyoto Encyclopedia of Genes and Genome), UniRef 90 [21], VFDB [22] (Virulence Factor Database), CARD [23] (Comprehensive Antibiotic Resistance Database), and CAZy [24] (Carbohydrate-Active enZYmes Database) were derived from the eggNOG-mapper results. We found that 44% of the nonredundant genes had a hit in at least one of the following databases: UniRef 90 (*n* = 69,720,696; 66.84%), eggNOG (*n* = 46,157,126; 44.25%), KEGG (*n* = 38,331,888; 36.75%), CAZy (*n* = 2,539,332; 2.43%), VFDB (*n* = 1,516,956; 1.45%), and CARD (*n* = 176,140; 0.17%) (Fig. 2B, C). After analyzing the annotated genes based on the eggNOG database, the predominant category was “Function unknown” (*n* = 5,776,948) (Fig. 2A). This category includes proteins that have not yet been characterized or for which there is insufficient information to assign a specific function. According to the eggNOG database annotation, 61.2% of the genes, including 58,056,469 unannotated genes and 5,776,948 genes labeled as “Function unknown,” were functionally unidentified, suggesting that mangrove sediments harbor numerous unknown functional genes.

**Figure 3:**
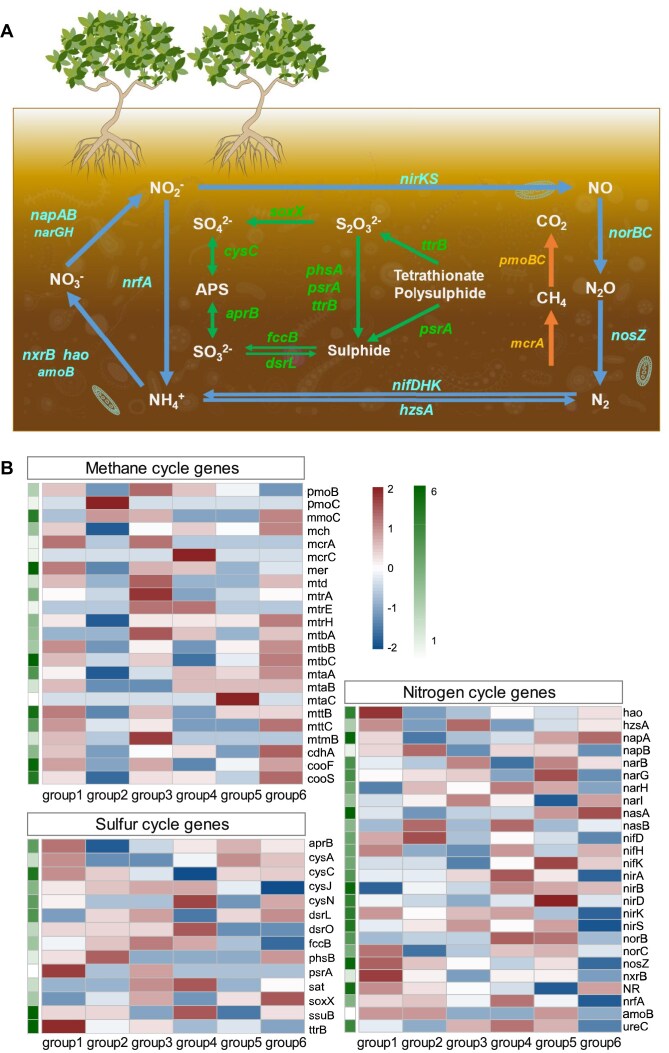
Primary metabolic processes and functional genes in the mangrove sediments. (A) Conceptual diagram of primary metabolic processes. The colors of the arrows represent the metabolic processes. Orange: methane cycle. Green: sulfur cycle. Blue: nitrogen cycle. (B) The abundance of genes implicated in methane, sulfur, and nitrogen cycles. Each row represents a gene, and each column represents a sampling group. For each gene, the total abundance of all samples was logarithmically transformed and shown on the right side (white-green block). The respective abundances of samples were logarithmically transformed and scaled by row (blue-red block).

To further understand the functional genes and pathways of methane (CH_4_), nitrogen (N), and sulfur (S) cycling microbiomes, we used MCycDB [25], SCycDB [26], and NCycDB [27] to calculate the abundances of functional genes involved in CH_4_, N, and S cycles in individual samples as well as in total samples (Fig. 3B). For CH_4_ cycling, the relatively abundant genes associated with the methane cycle in mangrove sediments included the genes *mcrAC, mtrAEH, mtd, mtaABC, mtbABC, mttBC, mtmB*, and *mch* for methanogenesis, as well as *pmoBC* and *mmoC* for methane oxidation. For N cycling, the representative genes were diverse and abundant in mangrove sediments, especially the genes *napAB, narGH, norBC*, and *nosZ* for denitrification; *nifDKH* for nitrogen fixation; *narB, NR, nasAB*, and *nirA* for assimilatory nitrate reduction; *nirBD* and *nrfA* for dissimilatory nitrate reduction; *hao* for nitrification; and *hzsA* and *nirKS* for anaerobic ammonium oxidation (anammox). For S cycling, the genes *cysCJN* for assimilatory sulfate reduction, *aprB* and *dsrLO* for dissimilatory sulfate reduction, *fccB* for sulfur oxidation, and *ttrB* and *soxX* for the SOX system were relatively abundant in mangrove sediments. The results suggested that, compared to other sampling groups, the genes of the methane cycle were more abundant in group 1 and group 6. In addition, except for *pmoC* and *mmoC*, lower abundances of other genes were detected in group 2, suggesting that the methane oxidation occurred to a lower extent in group 2. Besides, in group 6, the relative abundance of the genes related to nitrogen cycle were lower than those in other groups, especially the genes for denitrification, dissimilatory nitrate reduction, and anammox. Based on the major functional genes identified using MCycDB, SCycDB, and NCycDB, we inferred the roles of the microbes in C, N, and S cycling and depicted the biogeochemical transformations potentially driven by the microbes in mangrove sediments (Fig. 3A). For example, the microorganisms can realize the interconversion of CO_2_ and CH_4_. They can also facilitate the conversion between different valence states of N or S compounds.

### Phylogenomic analysis of archaeal and bacterial MAGs

**Figure 4:**
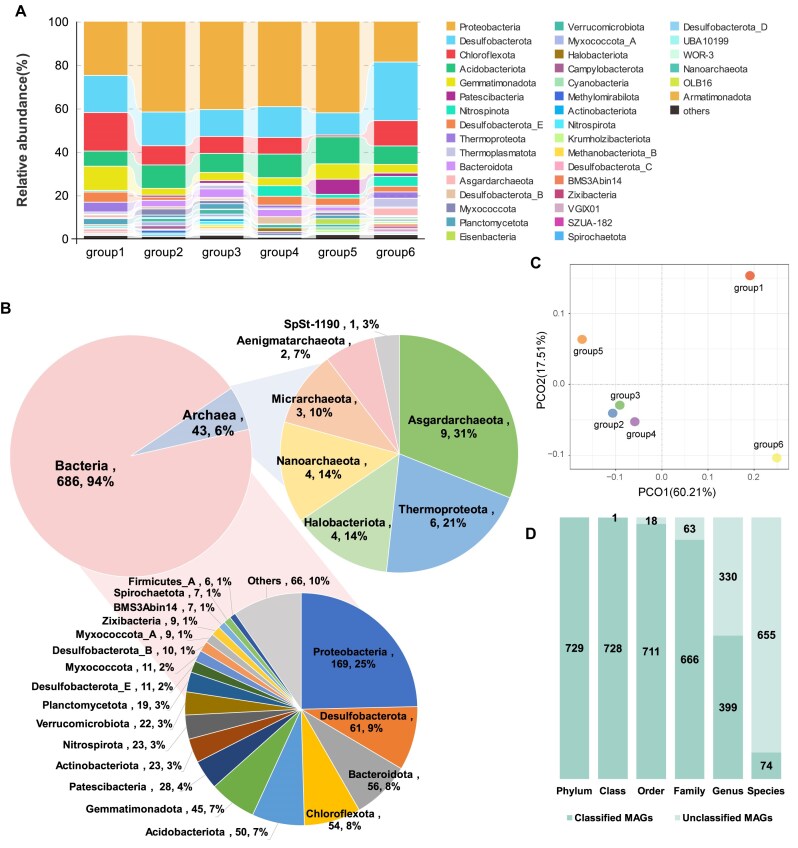
Taxonomic classification (domain and phylum levels) of the species-level representative MAGs. (A) The relative abundance of each archaeal or bacterial phylum, the coverage of each MAG was calculated using CoverM (version 0.6.1). (B) Genome statistics for the representative species of nonredundant MAGs. (C) The results of PCoA for the total microbial community in different sampling groups. (D) Taxonomic novelty of the representative species.

**Figure 5:**
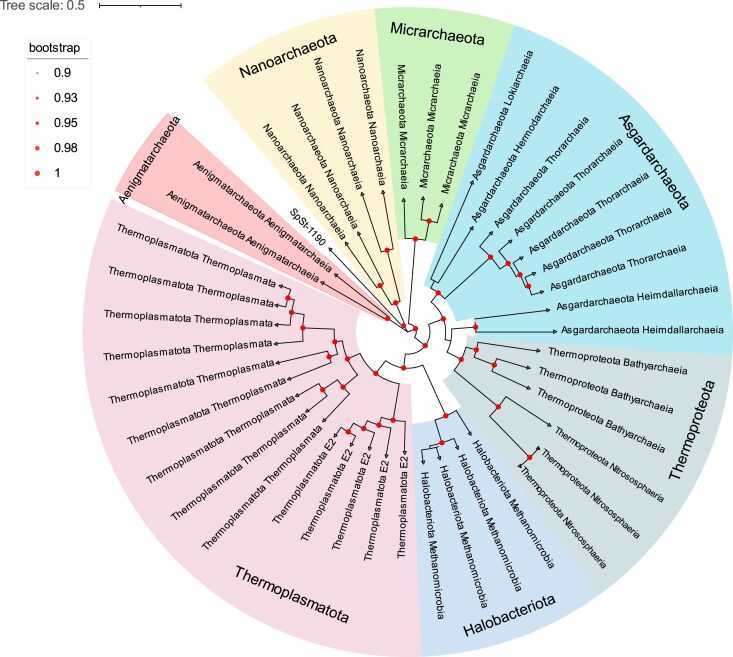
Phylogenomic analysis of archaeal MAGs. The phylogenetic tree was constructed from 43 MAGs from this study. The number of MAGs in each phylum is indicated in parentheses after the phylum name. The bootstrap values >0.9 are shown as red dots on nodes. The tree is unrooted.

**Figure 6:**
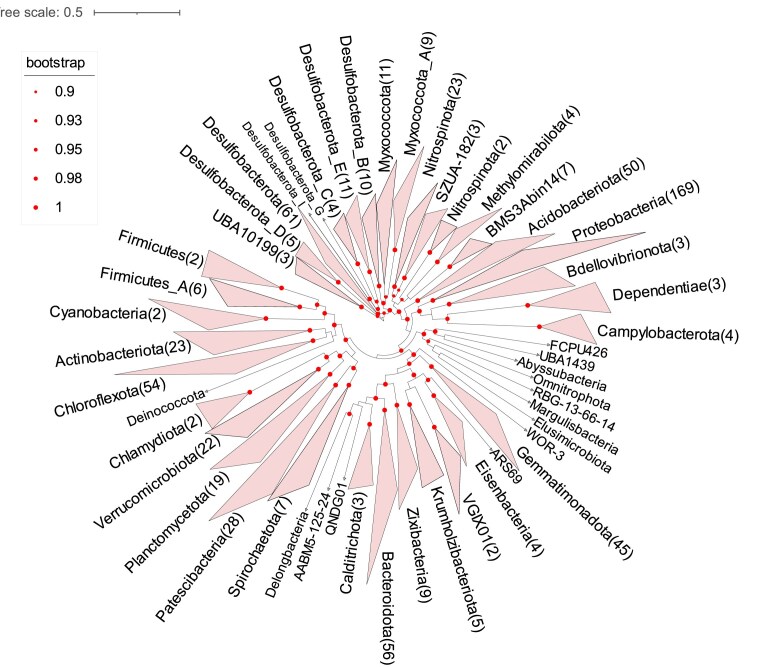
Phylogenomic analysis of bacterial MAGs. The phylogenetic tree was constructed from 686 MAGs from this study. The number of MAGs in each phylum is indicated in parentheses after the phylum name. The bootstrap values >0.9 are shown as red dots on nodes. The tree is unrooted.

MAGs were taxonomically classified using the GTDB-Tk toolkit (v2.1.1) [28, 29] with default parameters against the R207 database. Of the MAGs, 99.9% were annotated at the class level, 97.5% at the order level, 91.4% at the family level, 54.7% at the genus level, and 10.2% at the species level (Fig. 4D). Phylogenomic analysis based on single-copy marker genes showed that according to the taxonomy classification, 729 MSMs covered various prokaryotic lineages spanning 58 phyla (50 bacterial and 8 archaeal), 119 classes, 233 orders, 326 families, and 299 genera (Fig. 5 and Fig. 6). Mangrove sediment exhibited a higher relative abundance of Thermoplasmatota (0.7%–4.1%), Thermoproteota (0.3%–4.5%), and Asgardarchaeota (0.7%–3.5%) in archaea and Proteobacteria (24.8%–42.0%), Desulfobacterota (10.2%–27.0%), Chloroflexota (0.82%–17.7%), Acidobacteriota (7.0%–12.5%), and Gemmatimonadota (3.2%–11.2%) in bacteria (Fig. 4A). The community composition and abundance of species varied between different mangroves in China (Fig. 4A, C). The principal coordinate analysis (PCoA) results showed that the location of the mangroves had a significant influence on the microbial community (*P* < 0.05) (Fig. 4C). Concretely, Desulfobacterota was the species with the highest relative abundance in group 6 (27.0%), while Proteobacteria had the highest percentage in the other sampling groups (24.8%–42.0%). The relative abundance of Gemmatimonadota in group 1 (11.2%) was higher than the other groups (3.2%–7.1%). Group 5 had significantly less Chloroflexota (0.8%) and more abundant Patescibacteria (42.0%). The bacterial phyla with the largest diversity of recovered species included Proteobacteria (*n* = 169), Desulfobacterota (*n* = 61), Bacteroidota (*n* = 56), Chloroflexota (*n* = 54), Acidobacteriota (*n* = 50), and Gemmatimonadota (*n* = 45). The archaeal phyla with the largest diversity of recovered species included Thermoplasmatota (*n* = 14), Asgardarchaeota (*n* = 9), and Thermoproteota (*n* = 6) (Fig. 4B). There are 139 bacterial phyla and 21 archaea phyla in the GTDB, and the MSM dataset contains 36.0% of bacteria and 42.8% of archaea phyla of all prokaryotes, which shows that mangrove ecosystems have a high microbial diversity.

## Discussion

Mangroves, located in unique coastal and estuarine areas, are subjected to fluctuating conditions that are projected to escalate with climate change, underscoring the necessity for a deeper comprehension of the microbe–mangrove interactions [14]. Mangrove sediment microbiome is essential for the functioning and adaptability of mangrove ecosystems. The microbial landscape in mangrove sediment may influence nutrient cycling efficiency and be correlated with the distribution of key biological elements [30]. This is urgently needed for successful conservation and rehabilitation under changing conditions, making the nascent study of mangrove microbiome functions a high priority [17].

Given the pivotal role of mangrove ecosystems in coastal resilience and carbon sequestration, an urgent imperative emerges for a deeper understanding of their microbial communities. The intricate interplay between mangrove microbiomes and environmental parameters underscores the necessity for comprehensive investigations into microbial genomes within these unique habitats. Metagenome-assembled genomes are crucial for understanding microbial diversity, function, and ecology in various environments, including mangrove sediments. By sharing this database, researchers can gain insights into the unique metabolic adaptations of microbial communities in China mangrove sediments. This information can lead to discoveries of novel microbial lineages, metabolic pathways, and interactions within these ecosystems. This resource of 966 medium- and high-quality MAGs greatly complements the diversity of bacterial and archaeal genomic across global mangrove sediment biomes. The microbial genomes provided here suggest great biological diversity in mangrove ecosystems. Compared with other ecosystems, mangrove ecosystems significantly have higher microbial alpha diversity and microbial beta diversity [14]. The MSM catalog considerably expands the known phylogenetic diversity of bacteria and archaea, as well as increases recruitment of metagenomic sequencing reads.

Mangrove sediment ecosystems are extremely productive largely due to their efficient nutrient cycling systems for transformation of elements such as C, N, and S. Microorganisms likely play a significant role in element cycling in mangrove sediments. Bacteria dominate the microbial biomass, substantially influencing energy flow and nutrient cycling. Although the archaea were less abundant than bacteria, they are also important in mangrove ecosystems, since they can thrive in anaerobic and saline conditions, contributing to specific processes such as methane production and nitrogen fixation [31, 32]. In this study, the MSM catalog covered various prokaryotic lineages spanning 58 phyla, and the generally dominant phyla were Thermoplasmatota, Thermoproteota, and Asgardarchaeota in archaea and Proteobacteria, Desulfobacterota, Chloroflexota, Acidobacteriota, and Gemmatimonadota in bacteria (Fig. 4A). The distribution of these microorganisms varies in mangrove sediments from different sampling groups, which may be related to the local climate and the behaviors of other organisms in or surrounding mangrove ecosystems. In these phyla, Thermoplasmatota and Thermoproteota are known for their roles in anaerobic processes, particularly in the degradation of organic matter and the cycling of S and N in mangrove sediments [33]. Proteobacteria (groups like Alphaproteobacteria, Deltaproteobacteria, and Gammaproteobacteria), as dominant phyla in mangrove sediments, are associated with specific microbial groups such as sulfur-oxidizing and sulfur-reducing in nature [34–36]. Desulfobacterota includes significant sulfate-reducing bacteria (SRB) that thrive in the anoxic conditions, which are typical in mangrove environments. In this study, Desulfobacterota was the second most abundant bacterial phylum. They are crucial for the biogeochemical cycling of S, as they utilize sulfate as an electron acceptor, thereby facilitating the degradation of organic pollutants and contributing to the overall health of the sediment ecosystem [37, 38]. In addition, we recovered 8 methanogen MAGs in phyla Methylomirabilota and Methanobacteriota from mangrove sediments, which make contributions for methane production. Besides, we observed class Nitrososphaeria in mangrove sediments, which plays a significant role in the nitrogen cycle of mangrove sediments, primarily through its involvement in nitrification processes [39]. In summary, our analyses indicate that archaeal and bacterial communities in mangrove sediments are likely functionally interconnected, with predicted roles in nutrient cycling, organic matter degradation, and pollutant remediation. The metabolic diversity observed in these microorganisms probably contributes to maintaining ecosystem functions and could potentially enhance resilience against environmental stressors. However, these interpretations are derived from metagenomic predictions and homology-based annotations, which inherently carry limitations. For instance, incomplete MAGs and potential cross-contamination may affect the accuracy of functional inferences, particularly for underrepresented taxa or novel pathways. Furthermore, the absence of experimental validation precludes definitive conclusions about causal relationships between microbial activities and biogeochemical processes. Future studies integrating cultivation-based approaches, multiomics data, and *in situ* measurements will be critical to verify these hypotheses. Despite these limitations, the MAGs presented here provide a foundational resource for exploring microbial ecology in mangrove sediments and highlight candidate taxa and genes for targeted investigation.

With that said, MAGs from the MSM catalog, like other MAGs generated to date, have several limitations for users to be aware of, including undetected contamination, low contiguity, and incompleteness. Although these MAGs may be important placeholders for many new candidate species, we expect many could potentially be supplemented in the future by higher-quality MAGs or ultimately by whole-genome sequences from clonal isolates. We anticipate that the MSM catalog will become a valuable resource for future metabolic and genome-centric data mining and experimental validation. Furthermore, the shared database can aid in optimizing and evaluating the reconstruction of metagenome-assembled microbial genomes, overcoming database biases and enhancing the accuracy of genomic descriptions [40]. This optimization is essential for generating high-quality MAGs that reflect the true genetic potential of the microbial populations in the mangrove sediments. Additionally, researchers can leverage the database to explore hidden microbial diversity, phylogenetic markers, and metabolic functions within the MAGs [41, 42]. Understanding the functional potential of hard-to-culture microorganisms in the mangrove sediments can provide valuable insights into microbial ecology and metabolism, contributing to broader knowledge of microbial communities in these ecosystems [43]. Sharing the MAGs database can also contribute to the discovery of novel genes involved in the degradation of hydrocarbons and other environmental pollutants, as seen in studies on biofouled plastic fabrics [44]. This information is crucial for environmental remediation efforts and understanding the microbial mechanisms involved in biodegradation processes [45–47]. Moreover, the database can aid in estimating the completeness and redundancy of MAGs, providing researchers with valuable information on the quality and reliability of the assembled genomes [48]. This assessment is essential for ensuring the accuracy of downstream analyses and interpretations based on the metagenomic data.

In conclusion, the MAG database of mangrove sediment microbiomes from southeastern China provides a foundational resource for exploring microbial diversity and ecosystem functions in these critical coastal habitats. The MSM catalog, encompassing 966 MAGs with an average completeness of 75.6%, offers a framework to investigate taxonomic composition, metabolic potential, and roles in biogeochemical cycling (e.g., of CH_4_, N, and S). These data may facilitate comparative studies across mangrove ecosystems and contribute to broader efforts in environmental microbiology. However, several limitations must be acknowledged. The sampling scope was restricted to 6 nature reserves in China, which may limit generalizability to global mangrove ecosystems with distinct environmental conditions. In addition, MAGs generated from metagenomes are inherently fragmented and may contain chimeric contigs, potentially biasing taxonomic assignments and functional predictions. Furthermore, the inferred metabolic pathways rely on homology-based annotations, and their ecological relevance requires validation through complementary approaches such as metatranscriptomics, stable isotope probing, or cultivation. Future studies integrating multiomics data, expanded geographic sampling, and experimental validation will be essential to unravel the complex interactions between mangrove microbiomes and ecosystem dynamics. Despite these constraints, this study highlights the value of genomic resources in guiding conservation strategies for mangrove ecosystems under anthropogenic and climatic pressures.

## Methods

### Study sites and sediment sampling

We collected 82 sediment samples from 6 representative mangrove nature reserves in 5 provinces in southeastern China. These sites are as follows: Ximendao National Marine Reserve, XMD; Yunxiao Zhangjiangkou National Nature Reserve, YX; Shenzhen Futian National Nature Reserve, SZ; Leizhou Nature Reserve, LZ; Dongzhaigang National Nature Reserve, DZG; and Danzhou Xinyinggang Nature Reserve, DZ (Fig. 1B). The geographical locations of the 6 nature reserves are significantly different. XMD represents the most northern boundary where mangroves can survive. YX represents the most northern national mangrove reserve. SZ is the only national nature reserve located in the urban hinterland. LZ is located in the south end of the mainland of China. DZG is the first Chinese mangrove wetland included in the List of Wetlands of International Importance. DZ is located on the west coast of Hainan province. Latitudes and longitudes of the sampling sites were recorded using a GPS unit. Sediment samples were collected using a stainless-steel sampler (10 by 10 cm). At each sampling site, 2 depths were sampled corresponding to the surface (0 to 10 cm) and subsurface (10 to 20 cm). For each sediment type, 3 replicates were sampled, resulting in a total of 97 sediment samples. All samples were transferred on ice to the laboratory in 3 days. Sediment samples were separated into 2 sets. One of the sample sets was stored at −40°C before DNA extraction.

### DNA extraction and sequencing

Sediment genomic DNA was extracted from 0.3 g of the samples using a DNeasy PowerSoil kit (Qiagen) according to the manufacturer’s instructions. The quantity of the extracted DNA were examined using NanoDrop ND-2000c UV-Vis spectrophotometer (NanoDrop Technologies, RRID:SCR_018042). The final DNA concentration was quantified by a fluorescent method (Qubit@ 2.0 Fluorometer; Thermo Fisher Scientific, RRID:SCR_020553). The DNA samples were stored at −20°C and used for later molecular analysis. Sequencing libraries were generated using the TruSeq DNA PCR-Free Sample Preparation Kit following the manufacturer’s recommendations. Prepared library DNA concentrations were determined with a Qubit HS DNA assay, and libraries were run on a High Sensitivity DNA chip using the Agilent 2100 Bioanalyzer to determine library average insert sizes (RRID:SCR_018043). Last, the library was sequenced on a Illumina Hiseq 2500 platform (RRID:SCR_016383), and 250-bp paired-end reads were generated.

### Sequence quality check, assembly, and binning

The data were processed using KneadData (v0.8.0) [49] for quality control, which utilizes Trimmomatic to remove adaptor sequences and low-quality reads. Next, the data were divided into 6 groups according to the sampled location (Table 1). For each group, the trimmed reads were coassembled into contigs using MetaHipMer (v2.0.1) [50], which utilizes a Bruijn graph approach based on *k*-mers. The software employs increasing values of *k*-mer lengths (27, 37, 47, 57, 67, 77, 87, 97,107, 117, 127) and scaff–*k*-mer lengths (127, 37). The iterative process guarantees the production of high-quality assemblies. The generated contigs in each group longer than 2,000 bp were binned using multistrategy binning approaches. Briefly, the trimmed reads of each group were mapped against the corresponding coassembled contigs using BWA-MEM (v0.7.17, RRID:SCR_010910) [51]. Samtools (v1.7, RRID:SCR_002105) [52] was used to convert the output sam file to a bam file, for which the coverage was calculated. The obtained coverage file was used for the binning process using MetaBAT2 (v2.15, RRID:SCR_019134) [53] with 4 sets of parameters and VAMB (v3.0.3) [54] with 7 sets of parameters. The 11 sets of draft bins were then analyzed using Das Tool (v1.1.4) [55] to obtain the final optimized MAGs.

### MAG quality check and refinement

The completeness and contamination of MAGs were evaluated with CheckM2 (v1.0.1, RRID:SCR_016646) [56]. Based on these results, we selected 966 MAGs that were estimated to be at least 50% complete, with less than 10% contamination. As additional indicators of completeness, we identified tRNA genes using tRNAscan-SE (v2.0, RRID:SCR_008637) [57] and rRNA genes using Infernal (v1.1.2, RRID:SCR_011809) [58] with models from the Rfam database [59]. Based on these results, we found that 18 of the 966 MAGs were classified as high quality based on the MIMAG standard (≥90% completeness, ≤5% contamination, ≥18 tRNA genes and presence of 5S, 16S, and 23S rRNA genes), with the remaining classified as medium quality. These 966 medium- to high-quality MAGs constitute our final MSM catalog.

### Taxonomic annotation and phylogenomic analysis

Taxonomic classification of the final MAGs in each group and the phylogenomic tree of concatenated alignment were performed with the GTDB-Tk package (v2.2.6, Release 207_v2, RRID:SCR_019136) [60]. The archaeal and bacterial phylogenomic trees were visualized in the Interactive Tree of Life (iTOL, RRID:SCR_018174) [61]. The relative abundance of each MAG was calculated using CoverM in genome mode (v0.6.1) by mapping clean reads from the 644 metagenomes to all MAGs. PCoA was used to depict the community composition shift of the total microbial community among sampling groups based on the Bray–Curtis dissimilarity matrices at the genus level.

### Functional analysis of the MSM gene catalog

Putative protein-coding sequences (CDSs) of MAGs were predicted using Prodigal (v2.6.3, RRID:SCR_011936) [62]. The predicted CDSs were then clustered by MMseqs2 (RRID:SCR_022962) [63] with the parameters easy-linclust -e 0.001, –min-seq-id 0.95 and -c 0.80. The representative amino acid sequences from each cluster were functionally annotated using eggNOG-mapper (v2.1.9, RRID:SCR_021165; default parameters) [64]. The functional annotations, including those for eggnog 5.0 (RRID:SCR_002456) [19], KEGG (RRID:SCR_012773) [20], UniRef 90 (RRID:SCR_010646) [21], VFDB (RRID:SCR_007969) [22], CARD (RRID:SCR_023995) [23], and CAZy(RRID:SCR_012909) [24], were derived from the eggNOG-mapper results. MCycDB [25], SCycDB [26], and NCycDB [27], which are the specialized databases for the representative functional genes in CH_4_, S, and N cycling, were used for profiling of functional genes in 6 groups of samples. We selected 63 representative functional genes, including 23 for the methane cycle, 14 for the sulfur cycle, and 26 for the nitrogen cycle, to study the functional profiles. We calculated the total abundance of each gene and the respective abundances of each group of the samples. After natural log transformation, R and RStudio were used to create the heatmap with the pheatmap package (version 1.0.12, RRID:SCR_000432).

## Supplementary Material

giaf081_Authors_Response_To_Reviewer_Comments_Original_Submission

giaf081_Authors_Response_To_Reviewer_Comments_Revision_1

giaf081_GIGA-D-24-00229_Original_Submission

giaf081_GIGA-D-24-00229_Revision_1

giaf081_GIGA-D-24-00229_Revision_2

giaf081_Reviewer_1_Report_Original_Submission Ranjith Kumavath -- 8/1/2024

giaf081_Reviewer_1_Report_Revision_1 Ranjith Kumavath -- 12/24/2024

giaf081_Reviewer_2_Report_Original_Submission Learn Han Lee, Ph.D -- 9/9/2024

giaf081_Reviewer_2_Report_Revision_1 Learn Han Lee, Ph.D -- 1/21/2024

## Data Availability

All raw sequences from the current study have been deposited in NODE [65] under accession numbers OEP00000712, OEP00001343, OEP00001444, OEP00001474, OEP00001512, OEP00001839, OEP00001892, OEP00001920, and OEP00001984. The MAGs have been deposited in eLMSG under accession numbers LMSG_G000027425.1–LMSG_G000028852.1. The raw sequences have also been deposited to NCBI SRA and GenBank under the BioProject accession number: PRJNA1150796. All additional supporting data are available in the *GigaScience* repository, GigaDB [66].

## Abbreviations

bp: base pairs; CARD: Comprehensive Antibiotic Resistance Database; CAZy: Carbohydrate-Active enZYmes Database; eggNOG: evolutionary genealogy of genes: Non-supervised Orthologous; KEGG: Kyoto Encyclopedia of Genes and Genome; MAGs: metagenome-assembled genomes; Mb: megabase pairs; MMI: Mangrove Microbiome Initiative; MSMs: mangrove sediment metagenomes; VFDB: Virulence Factor Database.
