# Supplementary material for: A holistic genome dataset of bacteria and archaea of mangrove sediments
Source: Gigascience. 2025 Aug 12;14:giaf081. doi: 10.1093/gigascience/giaf081 (PMC12343073; doi:10.1093/gigascience/giaf081)
Supplement: giaf081_GIGA-D-24-00229_Revision_2 [file giaf081_giga-d-24-00229_revision_2.pdf]

GigaScience

A Holistic Genome Dataset of Bacteria and Archaea of Mangrove Sediments

--Manuscript Draft--

**Manuscript Number:**	GIGA-D-24-00229R2
**Full Title:**	A Holistic Genome Dataset of Bacteria and Archaea of Mangrove Sediments
**Article Type:**	Data Note
**Funding Information:**	National Natural Science Foundation of China (92051102, 32370055) — Prof. Yang Liu<br>National Natural Science Foundation of China (92251306, 32225003, 31970105) — Prof. Meng Li<br>National Natural Science Foundation of China (91951207) — Prof. Zhili He<br>National Natural Science Foundation of China (42303064) — Prof. Wu Qu<br>National Natural Science Foundation of China (32200099) — Doc. Huan Du<br>National Natural Science Foundation of China (92251307) — Prof. Guoqing Zhang<br>National Natural Science Foundation of China (32070108) — Doc. Jie Pan<br>Shenzhen Science and Technology Innovation Program (JCYJ20200109105010363) — Prof. Meng Li<br>Shenzhen Science and Technology Innovation Program (KCXFZ20201221173404012) — Prof. Cuijing Zhang<br>Shenzhen University 2035 Program for Excellent Research (2022B002) — Prof. Meng Li
**Abstract:**	Background: Mangroves are one of the most productive marine ecosystems with high ecosystem service value. The sediment microbial communities contribute to pivotal ecological functions in mangrove ecosystems. However, the study of mangrove sediment microbiomes is limited. Findings: Here, we applied metagenome sequencing analysis of microbial communities in mangrove sediments across southeast China from 2014 to 2020. This genome dataset includes 966 metagenome-assembled genomes with ≥50% completeness and ≤10% contamination generated from six groups of samples. Phylogenomic analysis and taxonomy classification show that mangrove sediments are inhabited by microbial communities with high species diversity. Thermoplasmatota, Thermoproteota, Asgardarchaeota in archaea and Proteobacteria, Desulfobacterota, Chloroflexota, Acidobacteriota, Gemmatimonadota in bacteria dominate the mangrove sediments across southeast China. Functional analyses suggest that the microbial communities may contribute to the carbon, nitrogen and sulfur cycling in mangrove sediments. Conclusions: These combined microbial genomes provide an important complement of global mangrove genome datasets and may serve as a foundational resource for enhancing our understanding of the compositions and functions of mangrove sediment microbiomes.
**Corresponding Author:**	Yang Liu<br>Institute for Advanced Study<br>Shenzhen, Guangdong CHINA
**Corresponding Author Secondary Information:**	
**Corresponding Author's Institution:**	Institute for Advanced Study

Powered by Editorial Manager® and ProduXion Manager® from Aries Systems Corporation

**Corresponding Author's Secondary Institution:**	
**First Author:**	Shijun Pan
**First Author Secondary Information:**	
**Order of Authors:**	Shijun Pan
	Huan Du
	Ruiqi Zheng
	Cuijing Zhang
	Jie Pan
	Xilan Yang
	Cheng Wang
	Xiaolan Lin
	Jinhui Li
	Wan Liu
	Haokui Zhou
	Xiaoli Yu
	Shuming Mo
	Guoqing Zhang
	Guoping Zhao
	Wu Qu
	Chengjian Jiang
	Yun Tian
	Zhili He
	Yang Liu
	Meng Li
**Order of Authors Secondary Information:**	
**Response to Reviewers:**	Dear Dr Zhang,<br><br>We appreciate the opportunity to revise our manuscript titled “A Holistic Genome Dataset of Bacteria and Archaea of Mangrove Sediments” for publication in Giga Science (GIGA-D-24-00229R1) and are grateful for the positive and constructive comments provided by the reviewers. We have studied reviewer’s comments carefully and have made revision which marked in blue in the paper. We have tried our best to revise our manuscript according to the comments. Attached please find the revised version, which we would like to submit for your kind consideration.<br><br>We would like to express our great appreciation again to you and reviewers your consideration of publishing our manuscript in this journal and for the comments on our paper. We are looking forward to hearing from you.<br><br>Yours sincerely,<br><br>Yang Liu<br>Leading contacts: Yang Liu & Meng Li<br>Institute for Advanced Study, Shenzhen University<br>Nanhai Ave 3688, Shenzhen, China, 518060<br>Email: yangliu@szu.edu.cn; limeng848@szu.edu.cn

Powered by Editorial Manager® and ProduXion Manager® from Aries Systems Corporation

Response to Reviewers' Comments :
Thank you very much for dealing with our manuscript entitled "A Holistic Genome Dataset of Bacteria and Archaea of Mangrove Sediments". Your comments are constructive and valuable. We have further revised our manuscript to make it more rigorous through systematic language revisions, transparent elaboration of research limitations, and prospects for future research directions. The revised manuscript with highlights on changes and a clean version are provided for your consideration. The point-by-point responses to all the comments are listed as follows:

Reviewer #1 (Comments for the Author):
The Manuscript has not fully convinced by the authors. So I regrets to inform you that, I can't able to review this MS again. It must be rejected based on wrong interpretation on the sequence data.

Response: We appreciate the reviewer's time and critical assessment of our manuscript. We acknowledge the concern regarding potential misinterpretations in the sequence data analysis and take this feedback with utmost seriousness. To address this, we have taken the following actions in the revised manuscript:

1. We rigorously revised the language in the manuscript, adjusted the discussion and conclusion of possible over-interpretation of data, and avoided absolute expressions that might imply causality. Examples include:
Abstract: We adjusted the sentence from "indicate the potential roles of microbial communities in helping to drive the carbon, nitrogen and sulfur cycling" to "suggest that the microbial communities may contribute to the carbon, nitrogen and sulfur cycling" (lines 50-52). Also, we adjusted the sentence from "should greatly facilitate our understanding of the compositions and functions of mangrove sediment microbiomes" to "may serve as a foundational resource for enhancing our understanding of the compositions and functions of mangrove sediment microbiomes" (lines 52-55).
Background: We adjusted "the data set of mangrove sediment microbiomes will be a very valuable resource, which will help us enhance the understanding of the composition, structure and function of mangrove sediment microbiomes" to "the dataset of mangrove sediment microbiomes represents a valuable resource, which could enhance our understanding of the composition, structure and putative functional roles of mangrove sediment microbiomes" (lines 76-78).
Data description: We clarified gene abundance trends as "relative differences" rather than definitive functional evidence (lines 151-153).
Discussion: We incorporated cautious modifiers (e.g., "may", "likely", "potentially") throughout the manuscript to frame hypotheses about microbial ecological roles, thereby avoiding overstatement of causal relationships. For example, assertions about nutrient cycling were revised from definitive claims (e.g., "influences", "the distribution of") to probabilistic language (e.g., "may influences", "be correlated with") in lines 192-194. In lines 214-215, we adjusted "one of the main drivers of the element cycling in mangrove sediments" to "likely play a significant role in element cycling in mangrove sediments". In lines 227-230, the phrase "drove the selection of" was revised to "are associated with" to reflect the correlative nature of metagenomic inferences. In lines 256-258, the phrase "will be replaced" was revised to "could potentially be supplemented" to more accurately reflect the provisional nature of MAGs (Metagenome-Assembled Genomes) while acknowledging that future advancements may enhance, rather than entirely replace these genomes.

2. We added an appropriate affirmation to the limitations of the dataset in our discussion and conclusion (lines 239-249 and 277-294), detailing the following limitations. To begin with, the mean completeness is 75.6% and the mean contamination rate is 4.1%, which may affect the accuracy of functional predictions, especially inference of low-abundance taxa or novel pathways (e.g., unannotated genes in the "Function unknown" category). Additionally, we acknowledged that the functional annotation relies on homology databases (e.g., KEGG, eggnog 5.0), which are inherently limited in annotating uncharacterized genes. Finally, we clarified that sampling focused on six reserves in southeastern China, limiting extrapolation to mangrove ecosystems with distinct environmental conditions (e.g., hypersaline estuaries or heavily polluted coastal zones).
Despite the limitations inherent to metagenome-assembled genomes (MAGs), this study provided three key contributions to mangrove microbiome research. First of all, it

Powered by Editorial Manager® and ProduXion Manager® from Aries Systems Corporation

	established a foundational MAG database spanning 58 prokaryotic phyla, significantly expanding the genomic representation of mangrove ecosystems globally. Secondly, it generated hypothesis-driven insights into critical biogeochemical processes, such as methane cycling (mcrA/pmoB genes) and sulfate reduction (dsrAB in Desulfobacterota), which prioritize targets for experimental validation via metatranscriptomics or stable isotope probing. While these MAGs are provisional placeholders, they may catalyze future work to resolve mangrove-microbe interactions through expanded sampling, multi-omics integration, and targeted cultivation of functionally critical taxa.<br><br>3. To validate ecological correlations among mangrove wetland microorganisms in future research, we proposed future validation directions to confirm ecological correlations among mangrove wetland microorganisms. Specifically, future studies should systematically integrate cultivation-based approaches, multi-omics analyses, and in situ measurement techniques to experimentally verify microbial metabolic pathways. (lines 249-253)<br><br>We are grateful for the opportunity to refine our work and hope that these changes meet with your approval. We welcome further guidance if specific analyses remain unclear.<br><br>Reviewer #2 (Comments for the Author):<br>The authors have done a good job in revising the work according to my comments. I hereby recommend the acceptance of the writing.<br><br>Response: Thank you for your positive feedback and constructive comments on our manuscript. We greatly appreciate your recognition of our revisions and your recommendation for acceptance. It is encouraging to know that our efforts to address the concerns raised in the review process have met your approval.
**Additional Information:**	
**Question**	**Response**
Are you submitting this manuscript to a special series or article collection?	No
**Experimental design and statistics**<br><br>Full details of the experimental design and statistical methods used should be given in the Methods section, as detailed in our Minimum Standards Reporting Checklist. Information essential to interpreting the data presented should be made available in the figure legends.<br><br>Have you included all the information requested in your manuscript?	Yes
**Resources**<br><br>A description of all resources used, including antibodies, cell lines, animals and software tools, with enough information to allow them to be uniquely	Yes

identified, should be included in the Methods section. Authors are strongly encouraged to cite Research Resource Identifiers (RRIDs) for antibodies, model organisms and tools, where possible.<br><br>Have you included the information requested as detailed in our Minimum Standards Reporting Checklist?	
**Availability of data and materials**<br><br>All datasets and code on which the conclusions of the paper rely must be either included in your submission or deposited in publicly available repositories (where available and ethically appropriate), referencing such data using a unique identifier in the references and in the "Availability of Data and Materials" section of your manuscript.<br><br>Have you have met the above requirement as detailed in our Minimum Standards Reporting Checklist?	Yes

Manuscript
Click here to access/download;Manuscript;Manuscript_June 17.docx

A Holistic Genome Dataset of Bacteria and Archaea of Mangrove Sediments

Authors

Shijun Pan[1,11,#], Huan Du[1,11,#], Ruiqi Zheng[9,10], Cuijing Zhang[1,11], Jie Pan[1,11], Xilan Yang[9,10], Cheng Wang[2,3], Xiaolan Lin[4], Jinhui Li[5], Wan Liu[7], Haokui Zhou[9,10], Xiaoli Yu[2,3], Shuming Mo[5], Guoqing Zhang[7], Guoping Zhao[7,8], Yang Liu[1,11,*], Meng Li[1,11,*]

Affiliations

1. Archaeal Biology Centre, Synthetic Biology Research Center, Shenzhen Key Laboratory of Marine Microbiome Engineering, Key Laboratory of Marine Microbiome Engineering of Guangdong Higher Education Institutes, Institute for Advanced Study, Shenzhen University, Shenzhen 518060, China

2. Southern Marine Science and Engineering Guangdong Laboratory (Zhuhai), Zhuhai 519080, China

3. State Key Laboratory for Biocontrol, School of Marine Sciences, School of Environmental Science and Engineering, Sun Yat-sen University, Guangzhou 510006, China

4. Key Laboratory of the Ministry of Education for Coastal and Wetland Ecosystems, School of Life Sciences, Xiamen University, Xiamen 361102, China

5. Guangxi Key Laboratory for Green Processing of Sugar Resources, College of Biological and Chemical Engineering, Guangxi University of Science and Technology, Liuzhou 545006, China.

6. Marine Science and Technology College, Zhejiang Ocean University, Zhoushan 316022, China

7. National Genomics Data Center& Bio-Med Big Data Center, CAS Key Laboratory of Computational Biology, Shanghai Institute of Nutrition and Health, University of Chinese Academy of Sciences, Chinese Academy of Science, Shanghai 200031, China

8. Hangzhou Institute for Advanced Study, University of Chinese Academy of Sciences, Hangzhou 310024, China

9. Shenzhen Key Laboratory of Synthetic Genomics, Guangdong Provincial Key Laboratory of Synthetic Genomics, CAS Key Laboratory of Quantitative Engineering Biology, Shenzhen 518055, China

10. Institute of Synthetic Biology, Shenzhen Institutes of Advanced Technology, Chinese Academy of Sciences, Shenzhen 518055, China

11. Shenzhen Key Laboratory of Marine Microbiome Engineering, Institute for Advanced Study, Shenzhen University, Shenzhen 518060, China.

[#] These authors contributed equally: Shijun Pan, Huan Du

corresponding author(s): Meng Li (limeng848@szu.edu.cn); Yang Liu (yangliu@szu.edu.cn).

Abstract

Background: Mangroves are one of the most productive marine ecosystems with high ecosystem service value. The sediment microbial communities contribute to pivotal ecological functions in mangrove ecosystems. However, the study of mangrove sediment microbiomes is limited. **Findings:** Here, we applied metagenome sequencing analysis of microbial communities in mangrove sediments across southeast China from 2014 to 2020. This genome dataset includes 966 metagenome-assembled genomes with ≥50% completeness and ≤10% contamination generated from six groups of samples. Phylogenomic analysis and taxonomy classification show that mangrove sediments are inhabited by microbial communities with high species diversity. Thermoplasmatota, Thermoproteota, Asgardarchaeota in archaea and Proteobacteria, Desulfobacterota, Chloroflexota, Acidobacteriota, Gemmatimonadota in bacteria dominate the mangrove sediments across southeast China. Functional analyses suggest that the microbial communities may contribute to the carbon, nitrogen and sulfur cycling in mangrove sediments. **Conclusions:** These combined microbial genomes provide an important complement of global mangrove genome datasets and may serve as a foundational resource for enhancing our understanding of the compositions and functions of mangrove sediment microbiomes.

Keywords

microbial composition, mangrove wetland, sediment microbiome, metagenome sequencing, metagenome-assembled genomes

Background

Mangroves are high-productivity ecosystems growing in swamp tidal areas of tropical and subtropical coastal areas. As an important part of coastal “blue carbon sink”, they play an extremely important role in purifying seawater, preventing waves, maintaining biodiversity and fixing carbon[1–5]. Mangroves provide a unique ecological environment. The special ecological characteristics, such as the dynamic change of salinity, large organic matter storage, anoxia and high redox potential value, create a high diversity and abundance of microbial community[6]. Similar to typical terrestrial plants, mangroves depend on mutually beneficial interaction with microbial communities[7]. The close relationship between microorganism, nutrient and plant promotes the cycle and preservation of main nutrients (carbon, nitrogen, phosphorus and sulfur), which is helpful to maintain the high productivity of mangrove ecosystem and further improve mangrove vegetation[8]. However, mangrove microbial community is threatened by global climate change and human factors such as coastal development, aquaculture and logging activities. It is urgent to take appropriate protection measures to deeply understand and protect this unique ecological community[9,10]. Although there have been many studies on mangrove ecosystem, the global exploration on microbial composition, distribution, interaction and ecological function of its sediments is still insufficient. Therefore, the dataset of mangrove sediment microbiomes represents a valuable resource, which could enhance our understanding of the composition, structure and putative functional roles of mangrove sediment microbiomes.

Mangroves in China are mainly distributed in Fujian, Zhejiang, Guangdong, Guangxi, Hainan, Hong Kong, Macao and Taiwan along the southeast coast, with a total area of about 28,900 hectares, accounting for 0.2% of the world. Due to the urbanization of coastal areas, the construction of ports and development zones, and the openness, fragility and complexity of mangrove ecosystem, its distribution is seriously affected by human activities[11,12]. For this

reason, China has established several mangrove nature reserves, which have alleviated the destruction and disappearance of mangroves to a certain extent, but the mangroves outside the reserves (often broken mangroves) have not improved significantly[13]. Although some ecological and genomic studies have been carried out on the microbial community of mangroves along the southeast coast of China[14–16], the comprehensive data set of microbial community including sediments of mangrove nature reserves needs to be improved. Acknowledging the existing gaps in our understanding, including limited studies on microbial communities within mangrove sediments and the fragmented nature of mangrove conservation efforts, collaborative initiatives such as the Mangrove Microbiome Initiative (MMI) have been established [17]. Through this international network of researchers, concerted efforts are being made to enhance the comprehensive dataset pertaining to microbial communities, especially within the sediments of mangrove nature reserves. This collaborative approach is crucial for advancing our understanding of microbial diversity, functionality, and evolutionary dynamics within mangrove ecosystems both in China and globally.

In this study, we deeply sequenced the deep-sequencing metagenomes of 6 groups of sediments collected in mangrove wetlands along the southeast coast of China from 2014 to 2020, spanning mangroves in six nature reserves (**Table 1** and **Fig. 1b**). We recover 966 medium and high-quality metagenome-assembled genomes (MAGs), which form the Genomes from the bacteria-focused and archaea-focused mangrove sediment metagenomes (MSMs) catalog (**Fig. 1a**). The MSM catalog was constructed from 644 metagenomes from theses mangrove natural reserves of China. The nonredundant gene and MSMs catalog are a valuable resource that will aid in deepening our understanding of the composition, structure and functions of mangrove sediment microbiomes.

Data description

Summary of reads, contigs and MSMs of mangrove sediment metagenomes

We performed metagenomic assembly and binning on 644 metagenomes from six mangrove natural reserves in five provinces, including Fujian (248), Guangxi (183), Guangdong (120),

Zhejiang (65) and Hainan provinces (28) (**Fig. 1b**). Overall, 7,216,630 contigs (> 2000 bp) were generated by assembling quality checked sequencing reads (**Table 1**). This catalog of MAGs contains representatives from all mangrove wetlands along the southeast coast of China. A total of 966 MAGs from the MSM catalog were reconstructed based on multi-strategy binning according to the MIMAG criteria[18] (mean completeness = 75.57%; mean contamination = 4.09%) and include 13 assigned as high quality based on the presence of a near-full complement of rRNAs, tRNAs and single-copy protein-coding genes (**Fig. 1a** and **Fig. 1c**). The genome sizes of these MSMs ranged from 0.26 to 7.76 Mb and GC content varied from 22% to 74%. Most small-sized MAGs belonging to the Aenigmatarchaeota, Nanoarchaeota or Patescibacteria, and similarly, large-sized MAGs belonging to Acidobacteriota or Desulfobacterota.

Table 1: Summary of reads, contigs and MAGs of MSMs.

Group	Read pairs after QC	Contigs (>2000bp)	Prokaryotic MAGs *
group1	589,800,176	572,210	117
group2	1,998,091,443	1,423,185	123
group3	4,271,704,109	3,743,050	296
group4	838,714,126	498,188	60
group5	1,392,266,226	447,022	41
group6	645,225,166	532,975	93

*Completeness ≥ 50%, contamination ≤ 10%.

Functional annotation and taxonomic classification of the MSM catalogs

The functional annotations, including those for eggnog 5.0[19] (evolutionary genealogy of genes:Non-supervised Orthologous), KEGG[20] (Kyoto Encyclopedia of Genes and Genome), UniRef 90[21], VFDB[22] (Virulence Factor Database), CARD[23] (Comprehensive Antibiotic Resistance Database) and CAZy[24] (Carbohydrate-Active enZYmes Database) were derived from the eggNOG-mapper results. We found that 44% of the non-redundant genes had a hit in at least one of the following databases: UniRef 90 (n = 69,720,696; 66.84%), eggNOG (n = 46,157,126; 44.25%), KEGG (n= 38,331,888; 36.75%), CAZy (n = 2,539,332; 2.43%), VFDB (n= 1,516,956; 1.45%) and CARD (n = 176,140; 0.17%) (**Fig. 2b** and **Fig. 2c**). After analyzing

the annotated genes based on the eggNOG database, the predominant category was "Function unknown" (n = 5,776,948) (**Fig. 2a**). This category includes proteins that have not yet been characterized or for which there is insufficient information to assign a specific function. According to the eggNOG database annotation, 61.2% of the genes, including 58,056,469 unannotated genes and 5,776,948 genes labeled as "Function unknown", were functionally unidentified, suggesting that mangrove sediments harbor numerous unknown functional genes.

To further understand the functional genes and pathways of methane (CH_4), nitrogen (N) and sulfur (S) cycling microbiomes, we used MCycDB[25], SCycDB[26], and NCycDB[27] to calculate the abundances of functional genes involving in CH_4, N and S cycles in individual samples as well as in total samples (**Fig. 3b**). For CH_4 cycling, the relatively abundant genes associated with methane cycle in mangrove sediments included the genes *mcrAC*, *mtrAEH*, *mtd*, *mtaABC*, *mtbABC*, *mttBC*, *mtmB*, *mch* for methanogenesis, and the genes *pmoBC*, *mmoC* for methane oxidation. For N cycling, the representative genes were diverse and abundant in mangrove sediments, especially the genes *napAB*, *narGH*, *norBC*, *nosZ* for denitrification, the genes *nifDKH* for nitrogen fixation, the genes *narB*, *NR*, *nasAB*, *nirA* for assimilatory nitrate reduction, the genes *nirBD*, *nrfA* for dissimilatory nitrate reduction, the genes *hao* for nitrification, and the genes *hzsA*, *nirKS* for anaerobic ammonium oxidation (anammox). For S cycling, the genes *cysCJN* for assimilatory sulfate reduction, the genes *aprB, dsrLO* for dissimilatory sulfate reduction, the genes *fccB* for sulfur oxidation and the genes *ttrB*, *soxX* for SOX system were relatively abundant in mangrove sediments. The results suggested that, compared to other sampling groups, the genes of methane cycle were more abundant in group1 and group6. In addition, except for *pmoC* and *mmoC*, lower abundances of other genes were detected in group2, suggesting that the methane oxidation occurred to a lower extent in group2. Besides, in group6, the relative abundance of the genes related to nitrogen cycle were lower than those in other groups, especially the genes for denitrification, dissimilatory nitrate reduction and anammox. Based on the major functional genes identified using MCycDB, SCycDB and NCycDB, we inferred the roles of the microbes in C, N and S cycling and depicted the biogeochemical transformations potentially driven by the microbes in mangrove sediments

(**Fig. 3a**). For example, the microorganisms can realize the interconversion of CO_2 and CH_4. They can also facilitate the conversion between different valence states of N or S compounds.

Phylogenomic analysis of archaeal and bacterial MAGs

MAGs were taxonomically classified using the GTDB-Tk toolkit (v2.1.1)[28] [29]with default parameters against the R207 database. 99.9% of MAGs were annotated at class level, 97.5% at order level, 91.4% at family level, 54.7% at genus level and 10.2% at species level (**Fig. 4d**). Phylogenomic analysis based on single-copy marker genes showed that according to the taxonomy classification, 729 MSMs covered various prokaryotic lineages spanning 58 phyla (50 bacterial and eight archaeal), 119 classes, 233 orders, 326 families and 299 genera (**Fig. 5** and **Fig. 6**). Mangrove sediment exhibited a higher relative abundance of Thermoplasmatota (0.7-4.1%), Thermoproteota (0.3-4.5%), Asgardarchaeota (0.7-3.5%) in archaea and Proteobacteria (24.8-42.0%), Desulfobacterota (10.2-27.0%), Chloroflexota (0.82-17.7%), Acidobacteriota (7.0-12.5%), Gemmatimonadota (3.2-11.2%) in bacteria (**Fig. 4a**). The community composition and abundance of species varied between different mangroves in China (**Fig. 4a and Fig. 4c**). The PCoA results showed that the location of the mangroves had a significant influence on the microbial community (p<0.05) (**Fig. 4c**). Concretely, Desulfobacterota was the species with the highest relative abundance in group6 (27.0%), while Proteobacteria had the highest percentage in the other sampling groups (24.8-42.0%). The relative abundance of Gemmatimonadota in group1 (11.2%) is higher than the other groups (3.2-7.1%). Group5 has significantly less Chloroflexota (0.8%) and more abundant Patescibacteria (42.0%). The bacterial phyla with the largest diversity of recovered species included Proteobacteria (n = 169), Desulfobacterota (n = 61), Bacteroidota (n= 56), Chloroflexota (n = 54), Acidobacteriota (n = 50), and Gemmatimonadota (n = 45). The archaeal phyla with the largest diversity of recovered species included Thermoplasmatota (n = 14), Asgardarchaeota (n = 9) and Thermoproteota (n = 6) (**Fig. 4b**). There are 139 bacterial phyla and 21 archaea phyla in GTDB, the MSM dataset contains 36.0% of bacteria and 42.8% of archaea phyla of all prokaryotes, which shows that mangrove ecosystems have a high microbial diversity.

Discussion

Mangroves located in unique coastal and estuarine areas, are subjected to fluctuating conditions that are projected to escalate with climate change, underscoring the necessity for a deeper comprehension of the microbe-mangrove interactions[14]. Mangrove sediment microbiome is essential for the functioning and adaptability of mangrove ecosystems. The microbial landscape in mangrove sediment may influences nutrient cycling efficiency and be correlated with the distribution of key biological elements[30]. This is urgently needed for successful conservation and rehabilitation under changing conditions, making the nascent study of mangrove microbiome functions a high priority[17].

Given the pivotal role of mangrove ecosystems in coastal resilience and carbon sequestration, an urgent imperative emerges for a deeper understanding of their microbial communities. The intricate interplay between mangrove microbiomes and environmental parameters underscores the necessity for comprehensive investigations into microbial genomes within these unique habitats. Metagenome-assembled genomes are crucial for understanding microbial diversity, function, and ecology in various environments, including mangrove sediments. By sharing this database, researchers can gain insights into the unique metabolic adaptations of microbial communities in China mangrove sediments. This information can lead to discoveries of novel microbial lineages, metabolic pathways, and interactions within these ecosystems. This resource of 966 medium- and high-quality MAGs greatly complements the diversity of bacterial and archaeal genomic across global mangrove sediment biomes. The microbial genomes provided here suggest great biological diversity in mangrove ecosystems. Compared with other ecosystems, mangrove ecosystems significantly have higher microbial alpha diversity and microbial beta diversity[14]. The MSMs catalog considerably expands the known phylogenetic diversity of bacteria and archaea, increases recruitment of metagenomic sequencing reads.

Mangrove sediment ecosystems are extremely productive largely due to their efficient nutrient cycling systems for transformation of elements such as C, N, and S. Microorganisms likely play a significant role in element cycling in mangrove sediments. Bacteria dominate the

microbial biomass, substantially influencing energy flow and nutrient cycling. Although the Archaea was less abundant than bacteria, they are also important in mangrove ecosystems, since they can thrive in the anaerobic and saline conditions, contributing to specific processes such as methane production and nitrogen fixation[31,32]. In this study, the MSM catalog covered various prokaryotic lineages spanning 58 phyla, and the generally dominant phyla were Thermoplasmatota, Thermoproteota, Asgardarchaeota in archaea and Proteobacteria, Desulfobacterota, Chloroflexota, Acidobacteriota, Gemmatimonadota in bacteria (**Fig. 4a**). The distribution of these microorganisms varies in mangrove sediments from different sampling groups, which may be related to the local climate and the behaviors of other organisms in or surrounding mangrove ecosystems. In these phyla, Thermoplasmatota and Thermoproteota are known for their roles in anaerobic processes, particularly in the degradation of organic matter and the cycling of S and N in mangrove sediments[33]. Proteobacteria (groups like Alphaproteobacteria, Deltaproteobacteria and Gammaproteobacteria), as dominant phyla in mangrove sediments, are associated with specific microbial groups such as sulphur-oxidizing and sulphur reducing in nature[34–36]. Desulfobacterota is significant sulfate-reducing bacteria (SRB) that thrive in the anoxic conditions which are typical in mangrove environments. In this study, Desulfobacterota was the second most abundant bacterial phylum. They are crucial for the biogeochemical cycling of S, as they utilize sulfate as an electron acceptor, thereby facilitating the degradation of organic pollutants and contributing to the overall health of the sediment ecosystem[37,38]. In addition, we recovered 8 methanogen MAGs in phyla Methylomirabilota and Methanobacteriota from mangrove sediments, which make contributions for methane production. Besides, we observed class Nitrososphaeria in mangrove sediments, which plays a significant role in the nitrogen cycle of mangrove sediments, primarily through its involvement in nitrification processes[39]. In summary, our analyses indicate that archaeal and bacterial communities in mangrove sediments are likely functionally interconnected, with predicted roles in nutrient cycling, organic matter degradation, and pollutant remediation. The metabolic diversity observed in these microorganisms probably contributes to maintaining ecosystem functions and could potentially enhance resilience against

environmental stressors. However, these interpretations are derived from metagenomic predictions and homology-based annotations, which inherently carry limitations. For instance, incomplete MAGs and potential cross-contamination may affect the accuracy of functional inferences, particularly for underrepresented taxa or novel pathways. Furthermore, the absence of experimental validation precludes definitive conclusions about causal relationships between microbial activities and biogeochemical processes. Future studies integrating cultivation-based approaches, multi-omics data, and *in situ* measurements will be critical to verify these hypotheses. Despite these limitations, the MAGs presented here provide a foundational resource for exploring microbial ecology in mangrove sediments and highlight candidate taxa and genes for targeted investigation.

With that said, MAGs from the MSMs catalog, like other MAGs generated to date, have several limitations for users to be aware of, including undetected contamination, low contiguity and incompleteness. Although these MAGs may be important placeholders for many new candidate species, we expect many could potentially be supplemented in the future by higher quality MAGs or ultimately by whole genome sequences from clonal isolates. We anticipate that the MSMs catalog will become a valuable resource for future metabolic and genome-centric data mining and experimental validation. Furthermore, the shared database can aid in optimizing and evaluating the reconstruction of metagenome-assembled microbial genomes, overcoming database biases and enhancing the accuracy of genomic descriptions[40]. This optimization is essential for generating high-quality MAGs that reflect the true genetic potential of the microbial populations in the mangrove sediments. Additionally, researchers can leverage the database to explore hidden microbial diversity, phylogenetic markers, and metabolic functions within the MAGs[41,42]. Understanding the functional potential of hard-to-culture microorganisms in the mangrove sediments can provide valuable insights into microbial ecology and metabolism, contributing to broader knowledge of microbial communities in these ecosystems[43]. Sharing the MAGs database can also contribute to the discovery of novel genes involved in the degradation of hydrocarbons and other environmental pollutants, as seen in studies on biofouled plastic fabrics[44]. This information is crucial for environmental

remediation efforts and understanding the microbial mechanisms involved in biodegradation processes[45–47]. Moreover, the database can aid in estimating the completeness and redundancy of MAGs, providing researchers with valuable information on the quality and reliability of the assembled genomes[48]. This assessment is essential for ensuring the accuracy of downstream analyses and interpretations based on the metagenomic data.

In conclusion, the MAG database of mangrove sediment microbiomes from southeastern China provides a foundational resource for exploring microbial diversity and ecosystem functions in these critical coastal habitats. The MSM catalog, encompassing 966 MAGs with an average completeness of 75.6%, offers a framework to investigate taxonomic composition, metabolic potential, and roles in biogeochemical cycling (e.g., of CH_4, N, and S). These data may facilitate comparative studies across mangrove ecosystems and contribute to broader efforts in environmental microbiology. However, several limitations must be acknowledged. The sampling scope was restricted to six nature reserves in China, which may limit generalizability to global mangrove ecosystems with distinct environmental conditions. In addition, MAGs generated from metagenomes are inherently fragmented and may contain chimeric contigs, potentially biasing taxonomic assignments and functional predictions. Furthermore, the inferred metabolic pathways rely on homology-based annotations, and their ecological relevance requires validation through complementary approaches such as metatranscriptomics, stable isotope probing, or cultivation. Future studies integrating multi-omics data, expanded geographic sampling, and experimental validation will be essential to unravel the complex interactions between mangrove microbiomes and ecosystem dynamics. Despite these constraints, this study highlights the value of genomic resources in guiding conservation strategies for mangrove ecosystems under anthropogenic and climatic pressures.

Methods

Study sites and sediment sampling. We collected 82 sediment samples from six representative mangrove nature reserves in five provinces in south-eastern China. These sites are as follows: Ximendao National Marine Reserve, XMD; Yunxiao Zhangjiangkou National Nature Reserve,

YX; Shenzhen Futian National Nature Reserve, SZ; Leizhou Nature Reserve, LZ; Dongzhaigang National Nature Reserve, DZG; Danzhou Xinyinggang Nature Reserve, DZ (**Fig. 1b**). The geographical locations of the six nature reserves are significantly different. XMD represents the most northern boundary where mangroves can survive. YX represents the most northern national mangrove reserve. SZ is the only national nature reserve located in the urban hinterland. LZ is located in the south end of the mainland of China. DZG is the first Chinese mangrove wetlands included in the List of Wetlands of International Importance. DZ is located in the west coast of Hainan province. Latitudes and longitudes of the sampling sites were recorded using a GPS unit. Sediment samples were collected using a stainless-steel sampler (10 cm by 10 cm). At each sampling site, two depths were sampled corresponding to the surface (0 to 10 cm) and subsurface (10 to 20 cm). For each sediment type, three replicates were sampled, resulting in a total of 97 sediment samples. All samples were transferred on ice to the laboratory in 3 days. Sediment samples were separated into two sets. One of the sample sets was stored at -40°C before DNA extraction.

DNA extraction and sequencing. Sediment genomic DNA was extracted from 0.3 g of the samples using a DNeasy PowerSoil kit (Qiagen, Germany) according to the manufacturer’s instructions. The quantity of the extracted DNA were examined using NanoDrop ND-2000c UV-Vis spectrophotometer (NanoDrop Technologies, Wilmington, DE, USA, RRID:SCR_018042). The final DNA concentration was quantified by a fluorescent method (Qubit@ 2.0 Fluorometer, Thermo Fisher Scientific, MA, USA, RRID:SCR_020553). The DNA samples were stored at -20°C and used for later molecular analysis. Sequencing libraries were generated using TruSeq® DNA PCR-Free Sample Preparation Kit following manufacturer's recommendations. Prepared library DNA concentrations were determined with a Qubit HS DNA assay and libraries were run on a High Sensitivity DNA chip using the Agilent 2100 Bioanalyzer to determine library average insert sizes (RRID:SCR_018043). At last, the library was sequenced on a Illumina Hiseq 2500 platform (RRID:SCR_016383) and 250 bp paired-end reads were generated.

Sequence quality check, assembly and bining. The data is processed using KneadData (v0.8.0)[49] for quality control, which utilizes Trimmomatic to remove adaptor sequences and low-quality reads. Next, the data were divided into six groups according to the sampled location (**Table 1**). For each group, the trimmed reads were co-assembled into contigs using MetaHipMer (v2.0.1)[50], which utilizes a Bruijn graph approach based on k-mers. The software employs increasing values of k-mer lengths (27, 37, 47, 57, 67, 77, 87, 97, 107, 117, 127) and scaff-kmer lengths (127, 37). The iterative process guarantees the production of high-quality assemblies. The generated contigs in each group longer than 2000 bp were binned using multi-strategy binning approaches. Briefly, the trimmed reads of each group were mapped against the corresponding co-assembled contigs using BWA-MEM(v0.7.17, RRID:SCR_010910)[51]. Samtools (v1.7, RRID:SCR_002105)[52] was used to convert the output sam file to bam file for which the coverage was calculated. The obtained coverage file was used for binning process using MetaBAT2(v2.15, RRID:SCR_019134)[53] with 4 sets of parameters and VAMB (v3.0.3)[54] with 7 sets of parameters. The 11 sets of draft bins were then analyzed using Das Tool (v1.1.4)[55] to obtain the final optimized metagenome-assembled genomes (MAGs).

MAG quality check and refinement. The completeness and contamination of MAGs were evaluated with CheckM2 (v1.0.1, RRID:SCR_016646)[56]. Based on these results, we selected 966 MAGs that were estimated to be at least 50% complete, with less than 10% contamination. As additional indicators of completeness, we identified tRNA genes using tRNAscan-SE (v2.0, RRID:SCR_008637)[57] and rRNA genes using Infernal (v1.1.2, RRID:SCR_011809)[58] with models from the Rfam database[59]. Based on these results, we found that 18 of the 966 MAGs were classified as high quality based on the MIMAG standard (≥90% completeness, ≤5% contamination, ≥18 tRNA genes and presence of 5S, 16S and 23S rRNA genes), with the remaining classified as medium quality. These 966 medium- to high-quality MAGs constitute our final MSM catalog.

Taxonomic annotation and phylogenomic analysis. The taxonomic classification of the MAGs was. Taxonomic classification of the final MAGs in each group and the phylogenomic tree of concatenated alignment were performed with the GTDB-Tk package (v2.2.6, Release 207_v2, RRID:SCR_019136)[60]. The archaeal and bacterial phylogenomic trees were visualized in the Interactive Tree of Life (iTOL, RRID:SCR_018174)[61]. The relative abundance of each MAG was calculated using CoverM in genome mode (v0.6.1) by mapping clean reads from the 644 metagenomes to all MAGs. Principal coordinate analysis (PCoA) was used to depict the community composition shift of total microbial community among sampling groups based on the Bray–Curtis dissimilarity matrices on the genus level.

Functional analysis of MSM gene catalog. And putative protein-coding sequences (CDSs) of MAGs were predicted using Prodigal (v2.6.3, RRID:SCR_011936)[62]. The predicted CDSs were then clustered by MMseqs2 (RRID:SCR_022962)[63] with the parameters easy-linclust -e 0.001, –min-seq-id 0.95 and -c 0.80, The representative amino acid sequences from each cluster were functionally annotated using eggNOG-mapper (v2.1.9, RRID:SCR_021165; default parameters)[64]. The functional annotations, including those for eggnog 5.0(RRID:SCR_002456) [19], KEGG (RRID:SCR_012773) [20], UniRef 90 (RRID:SCR_010646) [21], VFDB (RRID:SCR_007969) [22], CARD (RRID:SCR_023995) [23] and CAZy(RRID:SCR_012909) [24], were derived from the eggNOG-mapper results. MCycDB[25], SCycDB[26], and NCycDB[27], which are the specialized databases for the representative functional genes in CH_4, S, and N cycling, were used for profiling of functional genes in six groups of samples. We selected 63 representative functional genes, including 23 for the methane cycle, 14 for the sulfur cycle, and 26 for the nitrogen cycle, to study the functional profiles. We calculated the total abundance of each gene and the respective abundances of each group of the samples. After natural log transformation, R and RStudio were used to create the heatmap with the pheatmap package (version 1.0.12, RRID:SCR_000432).

Data Availability

All raw sequences from the current study have been deposited in NODE [65] under the accession numbers OEP00000712, OEP00001343, OEP00001444, OEP00001474, OEP00001512, OEP00001839, OEP00001892, OEP00001920, and OEP00001984. The MAGs have been deposited in eLMSG under the accession numbers LMSG_G000027425.1-LMSG_G000028852.1. The raw sequences have also been deposited to NCBI SRA and GenBank under the BioProject accession number: PRJNA1150796. All additional supporting data are available in the *GigaScience* repository, GigaDB [66].

Abbreviations

MMI: Mangrove Microbiome Initiative; MAGs: metagenome-assembled genomes; MSMs: mangrove sediment metagenomes; bp: base pairs; Mb: megabase pairs; eggNOG: evolutionary genealogy of genes: Non-supervised Orthologous; KEGG: Kyoto Encyclopedia of Genes and Genome; VFDB: Virulence Factor Database; CARD: Comprehensive Antibiotic Resistance Database; CAZy: Carbohydrate-Active enZYmes Database

Declarations

Not applicable

Consent for publication

Not applicable

Competing interests

The authors declare that they have no competing interests.

Funding

This work was financially supported by the Guangdong Major Project of Basic and Applied Basic Research (2023B0303000017), the National Natural Science Foundation of China (grant

nos. 92251306, 92051102, 92251307, 92351303, 32370055, 32200099, 32070108, 32225003, 31970105, 42303064 and 42430707), the Shenzhen Science and Technology Program (grant nos. JCYJ20200109105010363, KCXFZ20201221173404012, JCYJ20230808105711023, 20220809161641002), the Southern Marine Science and Engineering Guangdong Laboratory (Zhuhai) (SML2023SP218/SML2023SP237) and Shenzhen University 2035 Program for Excellent Research (2022B002).

Author contributions

Yang Liu and Meng Li conceived this study. Huan Du, Cuijing Zhang, Jie Pan, Xilan Yang, Cheng Wang, Xiaolan Lin, Jinhui Li, Xiaoli Yu and Shuming Mo conducted field sampling and DNA extraction. Shijun Pan, Huan Du, Ruiqi Zheng and Wan Liu assembled the metagenomes, generated the MAGs and conducted all other analyses. Shijun Pan and Huan Du produced all figures, interpreted the results and wrote the first draft. Yang Liu and Meng Li revised the draft. Haokui Zhou, Guoqing Zhang, Guoping Zhao provided computing resources and bioinformatics services. Wu Qu, Chengjian Jiang, Yun Tian, Zhili He and Meng Li provided data resources. All authors reviewed and contributed to the final version of the manuscript.

Acknowledgements

We would like to thank the collaborators in Bio-Med Big Data Center (Shanghai institute of Nutrition and Health, CAS), Xiamen University, Sun Yat-sen University, Guangxi University and Zhejiang Ocean University for sample collection and sequencing

Figures

Fig. 1 Geographical distribution of metagenome-assembled genomes. **(a)** A total of 966 MAGs were recovered from the mangrove sediment metagenomes. The majority of metagenomes were reassembled for this work using the latest state-of-the-art assembly pipeline. These genomes form the MSM catalog. All MAGs were ≥50% complete, were ≤5% contaminated. **(b)** Geographic distribution of the 6 groups of sediments' sites where metagenomic sequencing data

were collected. **(c)** Genome statistics for the representative species of non-redundant MAGs, showing the minimum value, first quartile, median, third quartile and maximum value.

Fig. 2 Functional annotation and taxonomic classification of the MSM catalog. **(a)** Functional annotations at the eggNOG category level. S: Function unknown. **(b)** An overview of annotations for the non-redundant gene catalog. Non-annotation indicates that these genes were not annotated in at least one of the following databases: eggNOG, KEGG, UniRef 90, VFDB, CARD and CAZy. **(c)** Number of genes with functional annotations across the six functional databases.

Fig. 3 Primary metabolic processes and functional genes in the mangrove sediments. **(a)** Conceptual diagram of primary metabolic processes. The colors of the arrows represent the metabolic processes. Orange: methane cycle. Green: sulfur cycle. Blue: nitrogen cycle. **(b)** The abundance of genes implicated in methane, sulfur, and nitrogen cycles. Each row represents a gene and each column represents a sampling group. For each gene, the total abundance of all samples was logarithmically transformed and shown on the right side (white-green block). The respective abundances of samples were logarithmically transformed and scaled by row (blue-red block).

Fig. 4 Taxonomic classification (domain and phylum levels) of the species-level representative MAGs. **(a)** The relative abundance of each archaeal or bacterial phyla, the coverage of each MAG was calculated using CoverM (version 0.6.1). **(b)** Genome statistics for the representative species of non-redundant MAGs. **(c)** The results of PCoA for the total microbial community in different sampling groups. **(d)**Taxonomic novelty of the representative species.

Fig. 5 Phylogenomic analysis of archaeal MAGs. The phylogenetic tree was constructed from 43 MAGs from this study. The number of MAGs in each phylum is indicated in parentheses after the phylum name. The bootstrap values > 0.9 are shown as red dots on nodes. The tree is unrooted.

Fig. 6 Phylogenomic analysis of bacterial MAGs. The phylogenetic tree was constructed from 686 MAGs from this study. The number of MAGs in each phylum is indicated in parentheses

after the phylum name. The bootstrap values > 0.9 are shown as red dots on nodes. The tree is unrooted.

References

1. Donato DC, Kauffman JB, Murdiyarso D, Kurnianto S, Stidham M, Kanninen M. Mangroves among the most carbon-rich forests in the tropics. *Nat Geosci*. Berlin: Nature Portfolio; 2011; doi: 10.1038/NGEO1123.
2. Moitinho MA, Chiaramonte JB, Bononi L, Gumiere T, Melo IS, Taketani RG. Fungal succession on the decomposition of three plant species from a Brazilian mangrove. *Sci Rep*. Nature Publishing Group; 2022; doi: 10.1038/s41598-022-18667-x.
3. Koch EW, Barbier EB, Silliman BR, Reed DJ, Perillo GM, Hacker SD, et al.. Non-linearity in ecosystem services: temporal and spatial variability in coastal protection. *Frontiers in Ecology and the Environment*. 2009; doi: 10.1890/080126.
4. Hochard JP, Hamilton S, Barbier EB. Mangroves shelter coastal economic activity from cyclones. *Proc Natl Acad Sci U S A*. 2019; doi: 10.1073/pnas.1820067116.
5. Temmerman S, Meire P, Bouma TJ, Herman PMJ, Ysebaert T, De Vriend HJ. Ecosystem-based coastal defence in the face of global change. *Nature*. 2013; doi: 10.1038/nature12859.
6. Kandasamy K, Bingham B. Biology of Mangroves and Mangrove Ecosystems. *Advances in Marine Biology*. 2001; doi: 10.1016/S0065-2881(01)40003-4.
7. Thatoi H, Behera BC, Mishra RR, Dutta SK. Biodiversity and biotechnological potential of microorganisms from mangrove ecosystems: a review. *Ann Microbiol*. 2013; doi: 10.1007/s13213-012-0442-7.
8. Holguin G, Vazquez P, Bashan Y. The role of sediment microorganisms in the productivity, conservation, and rehabilitation of mangrove ecosystems: an overview. *Biol Fertil Soils*. 2001; doi: 10.1007/s003740000319.
9. Palit K, Rath S, Chatterjee S, Das S. Microbial diversity and ecological interactions of microorganisms in the mangrove ecosystem: Threats, vulnerability, and adaptations. *Environ Sci Pollut Res Int*. 2022; doi: 10.1007/s11356-022-19048-7.
10. Lagomasino D, Fatoyinbo T, Castañeda-Moya E, Cook BD, Montesano PM, Neigh CSR, et al.. Storm surge and ponding explain mangrove dieback in southwest Florida following Hurricane Irma. *Nat Commun*. 2021; doi: 10.1038/s41467-021-24253-y.
11. Hagger V, Worthington TA, Lovelock CE, Adame MF, Amano T, Brown BM, et al.. Drivers of global mangrove loss and gain in social-ecological systems. *Nat Commun*. 2022; doi: 10.1038/s41467-022-33962-x.
12. Richards DR, Friess DA. Rates and drivers of mangrove deforestation in Southeast Asia, 2000–2012. *Proc Natl Acad Sci USA*. 2016; doi: 10.1073/pnas.1510272113.
13. Jia M, Wang Z, Zhang Y, Mao D, Wang C. Monitoring loss and recovery of mangrove forests during 42 years: The achievements of mangrove conservation in China. *International Journal of Applied Earth Observation and Geoinformation*. 2018; doi: 10.1016/j.jag.2018.07.025.
14. Zhang C-J, Pan J, Duan C-H, Wang Y-M, Liu Y, Sun 太阳 J, et al.. Prokaryotic Diversity in Mangrove Sediments across Southeastern China Fundamentally Differs from That in Other Biomes. *mSystems*. American Society for Microbiology; 2019; doi: 10.1128/mSystems.00442-19.
15. Zhuang W, Yu X, Hu R, Luo Z, Liu X, Zheng X, et al.. Diversity, function and assembly of mangrove root-associated microbial communities at a continuous fine-scale. *npj Biofilms Microbiomes*. 2020; doi: 10.1038/s41522-020-00164-6.
16. Wu P, Xiong X, Xu Z, Lu C, Cheng H, Lyu X, et al.. Bacterial Communities in the Rhizospheres of Three Mangrove Tree Species from Beilun Estuary, China. *PLOS ONE*. Public Library of Science; 2016; doi: 10.1371/journal.pone.0164082.

17. Allard SM, Costa MT, Bulseco AN, Helfer V, Wilkins LGE, Hassenrück C, et al.. Introducing the Mangrove Microbiome Initiative: Identifying Microbial Research Priorities and Approaches To Better Understand, Protect, and Rehabilitate Mangrove Ecosystems. *mSystems*. American Society for Microbiology; 2020; doi: 10.1128/msystems.00658-20.
18. Bowers RM, Kyrpides NC, Stepanauskas R, Harmon-Smith M, Doud D, Reddy TBK, et al.. Minimum information about a single amplified genome (MISAG) and a metagenome-assembled genome (MIMAG) of bacteria and archaea. *Nat Biotechnol*. 2017; doi: 10.1038/nbt.3893.
19. Huerta-Cepas J, Szklarczyk D, Heller D, Hernández-Plaza A, Forslund SK, Cook H, et al.. eggNOG 5.0: a hierarchical, functionally and phylogenetically annotated orthology resource based on 5090 organisms and 2502 viruses. *Nucleic Acids Research*. 2019; doi: 10.1093/nar/gky1085.
20. Kanehisa M, Furumichi M, Sato Y, Ishiguro-Watanabe M, Tanabe M. KEGG: integrating viruses and cellular organisms. *Nucleic Acids Research*. 2021; doi: 10.1093/nar/gkaa970.
21. Suzek BE, Wang Y, Huang H, McGarvey PB, Wu CH, the UniProt Consortium. UniRef clusters: a comprehensive and scalable alternative for improving sequence similarity searches. *Bioinformatics*. 2014; doi: 10.1093/bioinformatics/btu739.
22. Liu B, Zheng D, Zhou S, Chen L, Yang J. VFDB 2022: a general classification scheme for bacterial virulence factors. *Nucleic Acids Res*. 2022; doi: 10.1093/nar/gkab1107.
23. Alcock BP, Raphenya AR, Lau TTY, Tsang KK, Bouchard M, Edalatmand A, et al.. CARD 2020: antibiotic resistome surveillance with the comprehensive antibiotic resistance database. *Nucleic Acids Res*. 2020; doi: 10.1093/nar/gkz935.
24. Drula E, Garron M-L, Dogan S, Lombard V, Henrissat B, Terrapon N. The carbohydrate-active enzyme database: functions and literature. *Nucleic Acids Research*. 2022; doi: 10.1093/nar/gkab1045.
25. Qian L, Yu X, Zhou J, Gu H, Ding J, Peng Y, et al.. MCycDB: A curated database for comprehensively profiling methane cycling processes of environmental microbiomes. *Molecular Ecology Resources*. 2022; doi: 10.1111/1755-0998.13589.
26. Yu X, Zhou J, Song W, Xu M, He Q, Peng Y, et al.. SCycDB: A curated functional gene database for metagenomic profiling of sulphur cycling pathways. *Molecular Ecology Resources*. 2021; doi: 10.1111/1755-0998.13306.
27. Tu Q, Lin L, Cheng L, Deng Y, He Z. NCycDB: a curated integrative database for fast and accurate metagenomic profiling of nitrogen cycling genes. Wren J, editor. *Bioinformatics*. 2019; doi: 10.1093/bioinformatics/bty741.
28. Chaumeil P-A, Mussig AJ, Hugenholtz P, Parks DH. GTDB-Tk: a toolkit to classify genomes with the Genome Taxonomy Database. *Bioinformatics*. 2019; doi: 10.1093/bioinformatics/btz848.
29. Parks DH, Chuvochina M, Chaumeil P-A, Rinke C, Mussig AJ, Hugenholtz P. A complete domain-to-species taxonomy for Bacteria and Archaea. *Nat Biotechnol*. 2020; doi: 10.1038/s41587-020-0501-8.
30. Booth JM, Fusi M, Marasco R, Daffonchio D. The microbial landscape in bioturbated mangrove sediment: A resource for promoting nature-based solutions for mangroves. *Microbial Biotechnology*. 2023; doi: 10.1111/1751-7915.14273.
31. Zhang C-J, Chen Y-L, Sun Y-H, Pan J, Cai M-W, Li M. Diversity, metabolism and cultivation of archaea in mangrove ecosystems. *Mar Life Sci Technol*. 2021; doi: 10.1007/s42995-020-00081-9.
32. Liu M, Huang H, Bao S, Tong Y. Microbial community structure of soils in Bamenwan mangrove wetland. *Sci Rep*. 2019; doi: 10.1038/s41598-019-44788-x.
33. Maltseva AI, Klyukina AA, Elcheninov AG, Pimenov NV, Rusanov II, Kublanov IV, et al.. Water and Sediments of an Acidic Hot Spring—Distinct Differentiation with Regard to the Microbial Community Composition and Functions. *Water*. 2023; doi: 10.3390/w15193415.

34. Sanka Loganathachetti D, Sadaiappan B, Poosakkannu A, Muthuraman S. Pyrosequencing-Based Seasonal Observation of Prokaryotic Diversity in Pneumatophore-Associated Soil of Avicennia marina. *Curr Microbiol*. 2016; doi: 10.1007/s00284-015-0920-9.

35. Alzubaidy H, Essack M, Malas TB, Bokhari A, Motwalli O, Kamanu FK, et al.. Rhizosphere microbiome metagenomics of gray mangroves (Avicennia marina) in the Red Sea. *Gene*. 2016; doi: 10.1016/j.gene.2015.10.032.

36. Padhy SR, Bhattacharyya P, Nayak SK, Dash PK, Mohapatra T. A unique bacterial and archaeal diversity make mangrove a green production system compared to rice in wetland ecology: A metagenomic approach. *Sci Total Environ*. 2021; doi: 10.1016/j.scitotenv.2021.146713.

37. Paingankar MS, Deobagkar DD. Pollution and Environmental Stressors Modulate the Microbiome in Estuarine Mangroves:A Metagenome Analysis. *Current Science*. 2018; doi: 10.18520/cs/v115/i8/1525-1535.

38. Dos Santos HF, Cury JC, Do Carmo FL, Dos Santos AL, Tiedje J, Van Elsas JD, et al.. Mangrove Bacterial Diversity and the Impact of Oil Contamination Revealed by Pyrosequencing: Bacterial Proxies for Oil Pollution. Heimesaat M, editor. *PLoS ONE*. 2011; doi: 10.1371/journal.pone.0016943.

39. Zvi-Kedem T, Lalzar M, Sun J, Li J, Tchernov D, Meron D. Exploring the Microbial Mosaic: Insights into Composition, Diversity, and Environmental Drivers in the Pearl River Estuary Sediments. Microorganisms. 2024. doi: 10.3390/microorganisms12071273.

40. Papudeshi B, Haggerty JM, Doane M, Morris MM, Walsh K, Beattie DT, et al.. Optimizing and evaluating the reconstruction of Metagenome-assembled microbial genomes. *BMC Genomics*. 2017; doi: 10.1186/s12864-017-4294-1.

41. Lapidus AL, Korobeynikov AI. Metagenomic Data Assembly – The Way of Decoding Unknown Microorganisms. *Front Microbiol*. 2021; doi: 10.3389/fmicb.2021.613791.

42. Brown CT, Moritz D, O'Brien MP, Reidl F, Reiter T, Sullivan BD. Exploring neighborhoods in large metagenome assembly graphs using spacegraphcats reveals hidden sequence diversity. *Genome Biol*. 2020; doi: 10.1186/s13059-020-02066-4.

43. Sangwan N, Xia F, Gilbert JA. Recovering complete and draft population genomes from metagenome datasets. *Microbiome*. 2016; doi: 10.1186/s40168-016-0154-5.

44. . Metagenome-Assembled Genomes of 12 Bacterial Species from Biofouled Plastic Fabrics Harbor Multiple Genes for Degradation of Hydrocarbons. *Microbiology Resource Announcements*. 2021; doi: 10.1128/mra.01458-20.

45. Roy S, Hens D, Biswas D, Biswas D, Kumar R. Survey of petroleum-degrading bacteria in coastal waters of Sunderban Biosphere Reserve. *World Journal of Microbiology and Biotechnology*. 18:575–812002;

46. Chen J, Wang C, Shen Z-J, Gao G-F, Zheng H-L. Insight into the long-term effect of mangrove species on removal of polybrominated diphenyl ethers (PBDEs) from BDE-47 contaminated sediments. *Science of The Total Environment*. 2017; doi: 10.1016/j.scitotenv.2016.10.040.

47. Jiang Y, Lu H, Xia K, Wang Q, Yang J, Hong H, et al.. Effect of mangrove species on removal of tetrabromobisphenol A from contaminated sediments. *Chemosphere*. 2020; doi: 10.1016/j.chemosphere.2019.125385.

48. Hugoson E, Lam WT, Guy L. miComplete: weighted quality evaluation of assembled microbial genomes. *Bioinformatics*. 2020; doi: 10.1093/bioinformatics/btz664.

49. McIver LJ, Abu-Ali G, Franzosa EA, Schwager R, Morgan XC, Waldron L, et al.. bioBakery: a meta'omic analysis environment. *Bioinformatics*. 2018; doi: 10.1093/bioinformatics/btx754.

50. Hofmeyr S, Egan R, Georganas E, Copeland AC, Riley R, Clum A, et al.. Terabase-scale metagenome coassembly with MetaHipMer. *Sci Rep*. 2020; doi: 10.1038/s41598-020-67416-5.

51. Li H. Aligning sequence reads, clone sequences and assembly contigs with BWA-MEM. arXiv. 2013. https://doi.org/10.48550/arXiv.1303.3997.

52. Danecek P, Bonfield JK, Liddle J, Marshall J, Ohan V, Pollard MO, et al.. Twelve years of SAMtools and BCFtools. *GigaScience*. 2021; doi: 10.1093/gigascience/giab008.

53. Kang DD, Li F, Kirton E, Thomas A, Egan R, An H, et al.. MetaBAT 2: an adaptive binning algorithm for robust and efficient genome reconstruction from metagenome assemblies. *PeerJ*. PeerJ Inc.; 2019; doi: 10.7717/peerj.7359.

54. Nissen JN, Johansen J, Allesøe RL, Sønderby CK, Armenteros JJA, Grønbech CH, et al.. Improved metagenome binning and assembly using deep variational autoencoders. *Nat Biotechnol*. 2021; doi: 10.1038/s41587-020-00777-4.

55. Sieber CMK, Probst AJ, Sharrar A, Thomas BC, Hess M, Tringe SG, et al.. Recovery of genomes from metagenomes via a dereplication, aggregation and scoring strategy. *Nat Microbiol*. Nature Publishing Group; 2018; doi: 10.1038/s41564-018-0171-1.

56. Chklovski A, Parks DH, Woodcroft BJ, Tyson GW. CheckM2: a rapid, scalable and accurate tool for assessing microbial genome quality using machine learning. *Nat Methods*. Nature Publishing Group; 2023; doi: 10.1038/s41592-023-01940-w.

57. Lowe TM, Eddy SR. tRNAscan-SE: a program for improved detection of transfer RNA genes in genomic sequence. *Nucleic Acids Res*. 1997; doi: 10.1093/nar/25.5.955.

58. Nawrocki EP, Kolbe DL, Eddy SR. Infernal 1.0: inference of RNA alignments. *Bioinformatics*. 2009; doi: 10.1093/bioinformatics/btp157.

59. Kalvari I, Argasinska J, Quinones-Olvera N, Nawrocki EP, Rivas E, Eddy SR, et al.. Rfam 13.0: shifting to a genome-centric resource for non-coding RNA families. *Nucleic Acids Res*. 2018; doi: 10.1093/nar/gkx1038.

60. Chaumeil P-A, Mussig AJ, Hugenholtz P, Parks DH. GTDB-Tk v2: memory friendly classification with the genome taxonomy database. Borgwardt K, editor. *Bioinformatics*. 2022; doi: 10.1093/bioinformatics/btac672.

61. Letunic I, Bork P. Interactive Tree Of Life (iTOL) v5: an online tool for phylogenetic tree display and annotation. *Nucleic Acids Research*. 2021; doi: 10.1093/nar/gkab301.

62. Hyatt D, Chen G-L, LoCascio PF, Land ML, Larimer FW, Hauser LJ. Prodigal: prokaryotic gene recognition and translation initiation site identification. *BMC Bioinformatics*. 2010. doi: 10.1186/1471-2105-11-119.

63. Mirdita M, Steinegger M, Breitwieser F, Söding J, Levy Karin E. Fast and sensitive taxonomic assignment to metagenomic contigs. Kelso J, editor. *Bioinformatics*. 2021; doi: 10.1093/bioinformatics/btab184.

64. Cantalapiedra CP, Hernández-Plaza A, Letunic I, Bork P, Huerta-Cepas J. eggNOG-mapper v2: Functional Annotation, Orthology Assignments, and Domain Prediction at the Metagenomic Scale. *Molecular Biology and Evolution*. 2021; doi: 10.1093/molbev/msab293.

65. LING Yun-Chao, CAO Rui-Fang, LI Yi-Xue, ZHANG Guo-Qing. Advances in multi-omics big data sharing platform research. *Chinese Bulletin of Life Sciences*. 2023; doi: 10.13376/j.cbls/2023169.

66. Pan S, Du H, Zheng R, Zhang C, Pan J, Yang X, et al. Supporting data for "A Holistic Genome Dataset of Bacteria and Archaea of Mangrove sediments" GigaScience Database. 2025. https://doi.org/10.5524/102702.

Click here to access/download;Figure;Figures1.pdf

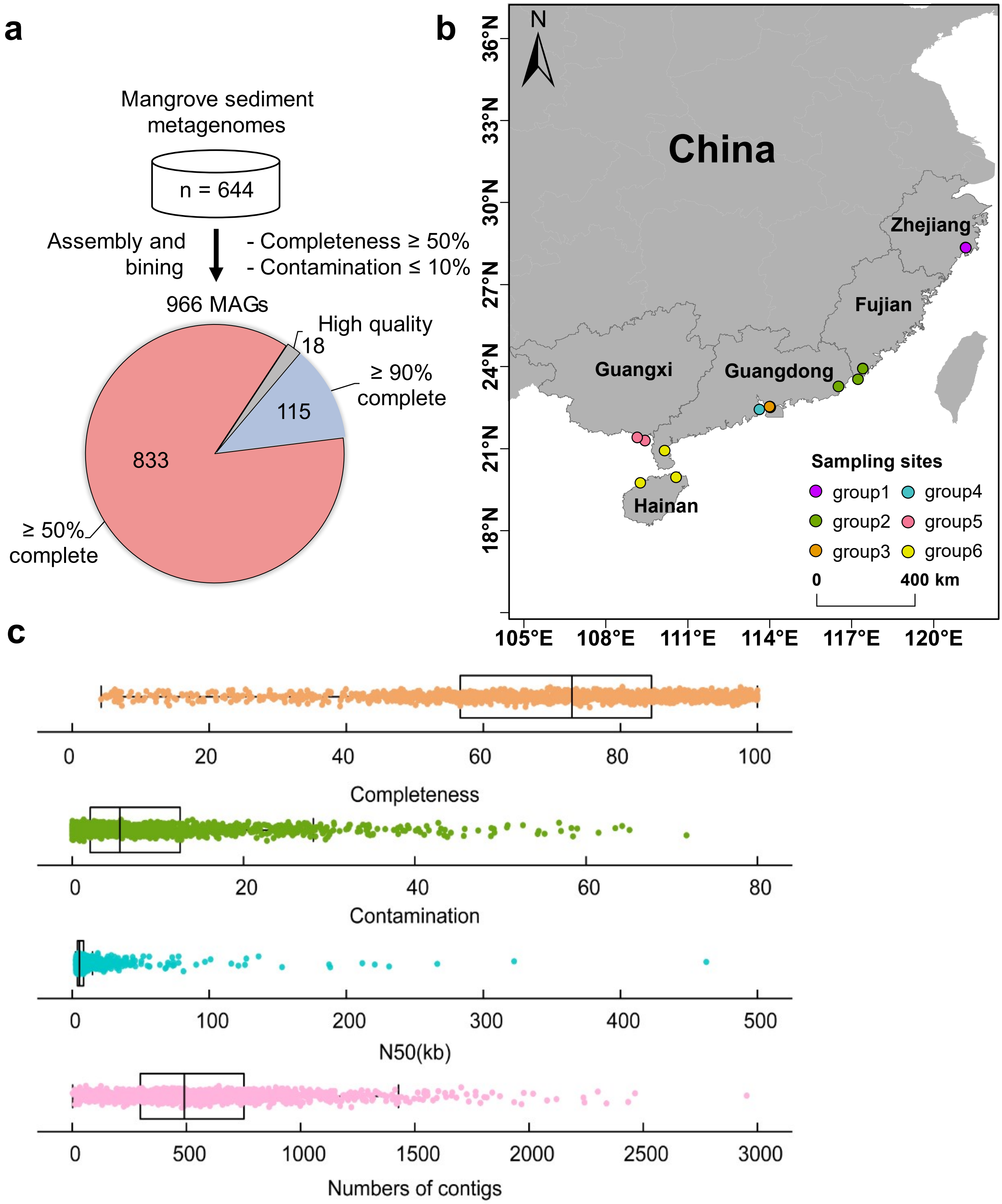

Fig. 1

 Click here to access/download;Figure;Figures2.pdf

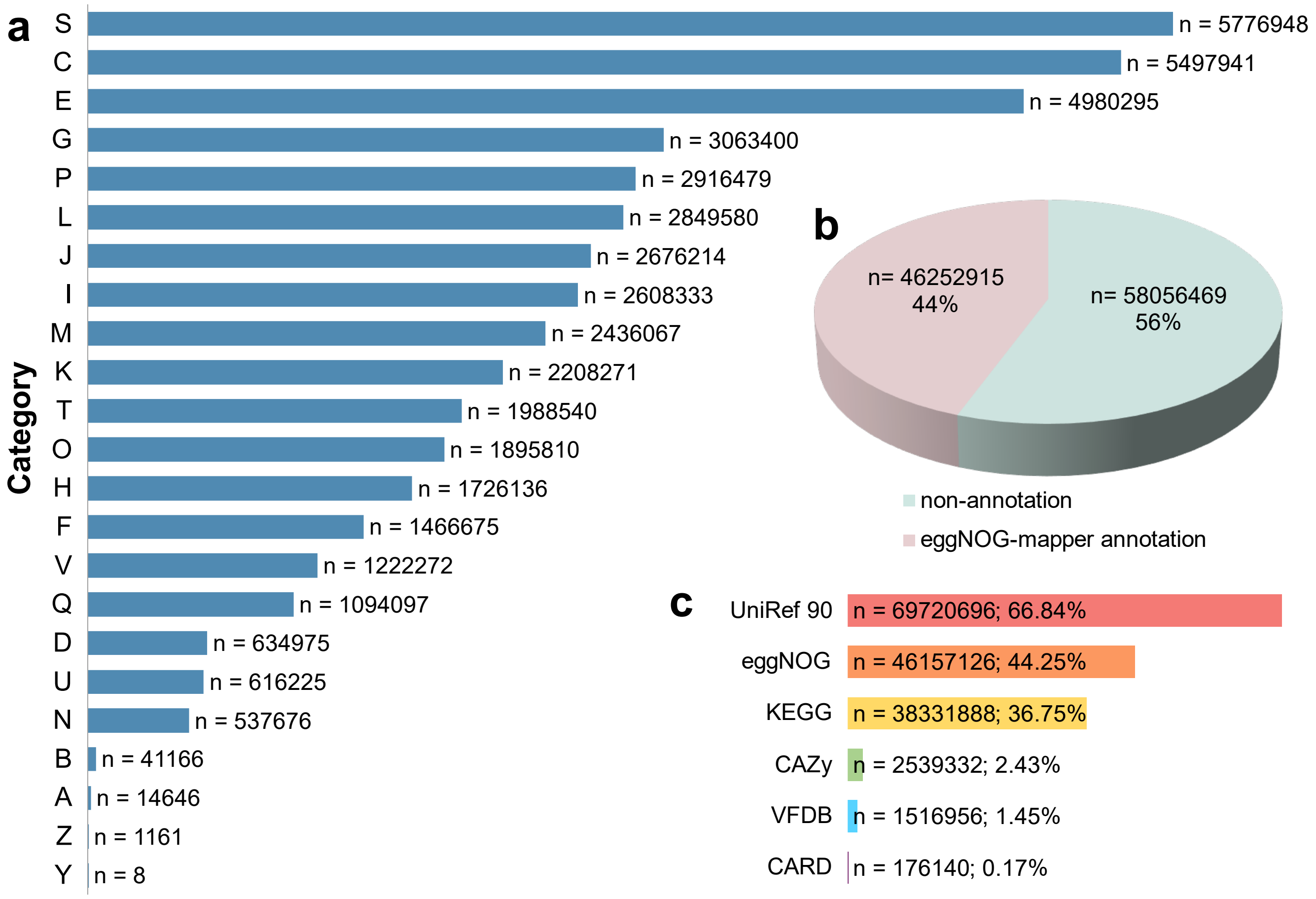

Fig. 2

Figure3

Click here to access/download;Figure;Figures3.pdf

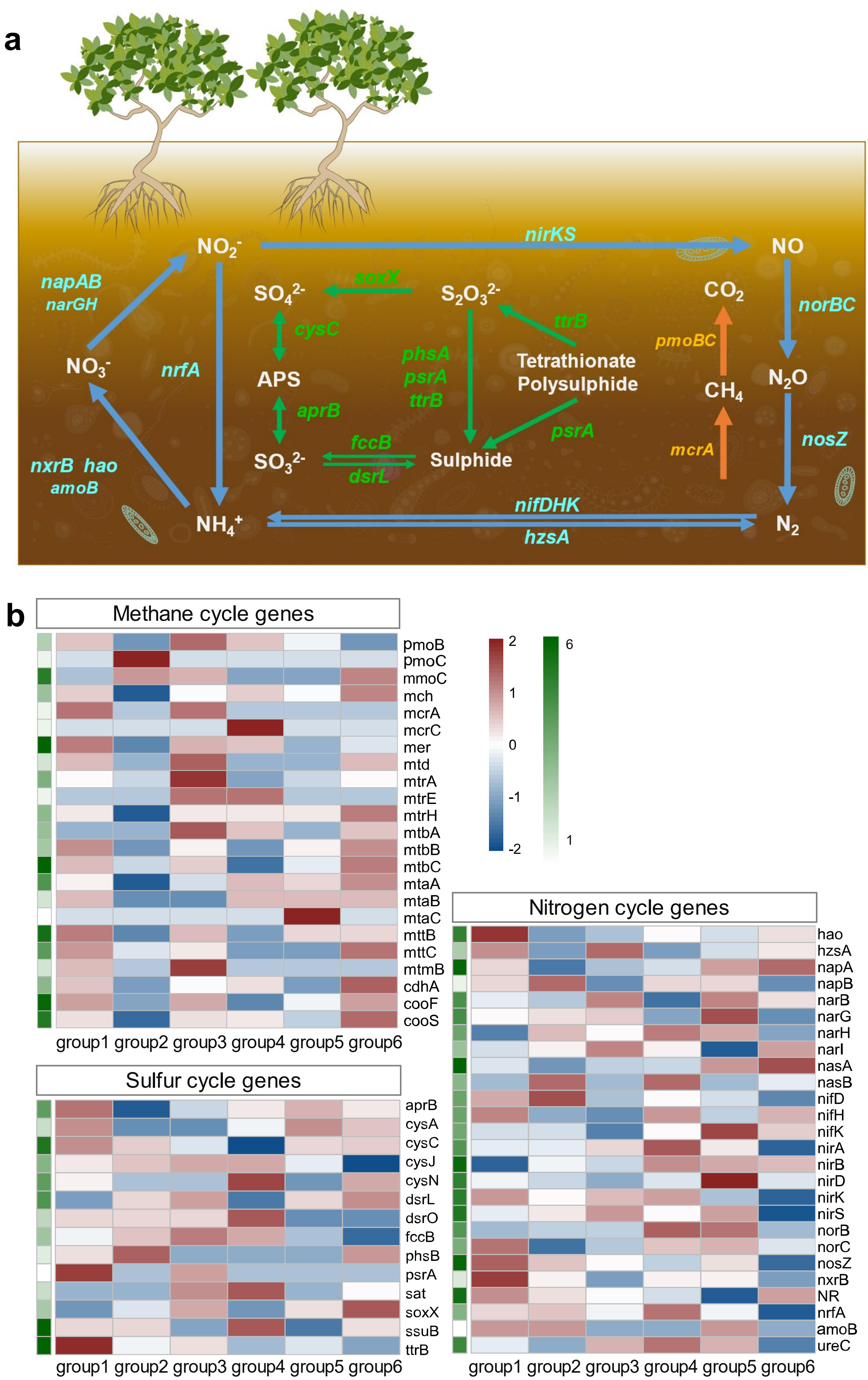

Fig. 3

Figure4 Click here to access/download;Figure;Figures4.pdf

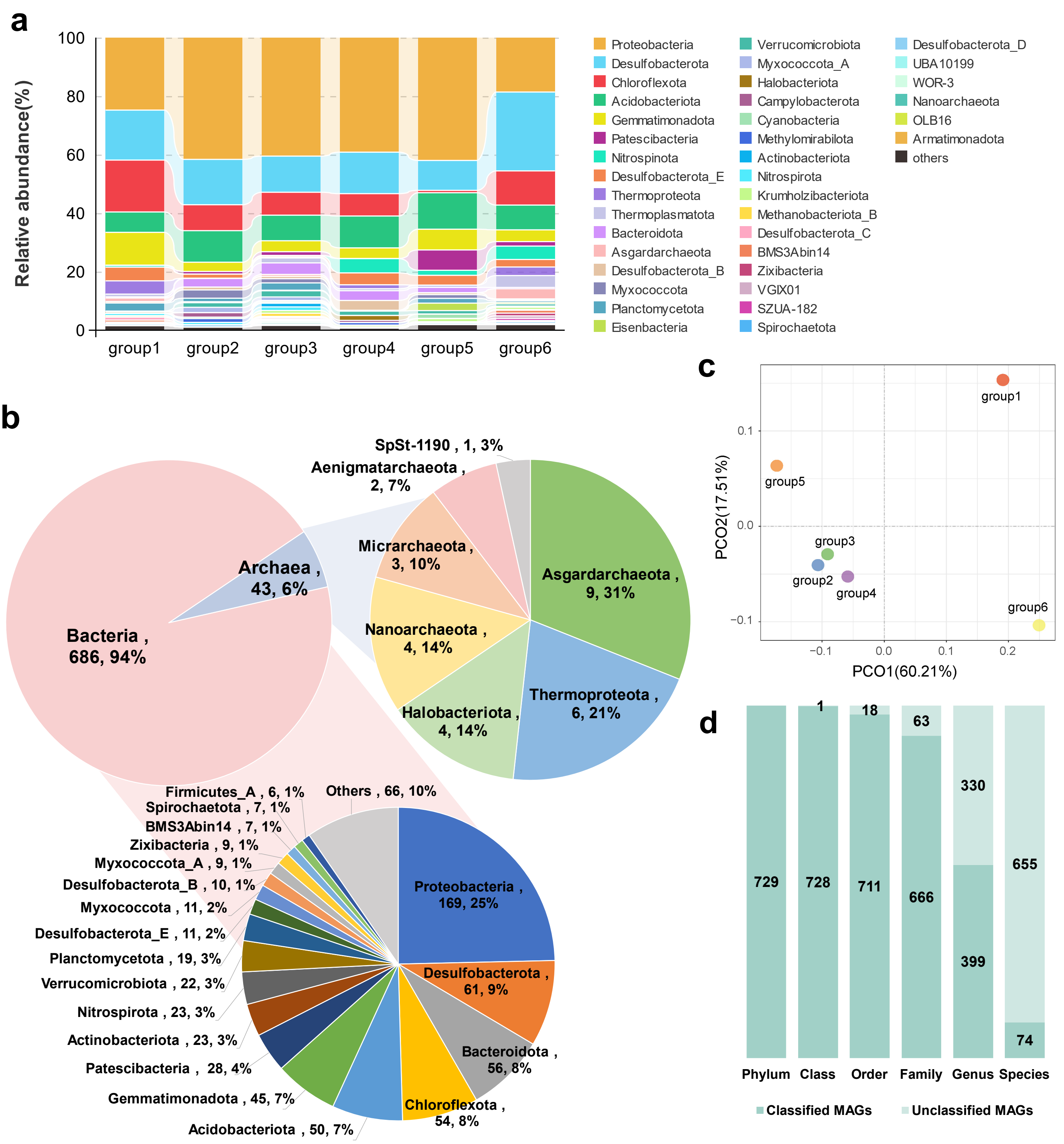

Fig. 4

Figure5

Click here to access/download;Figure;Figures5.pdf

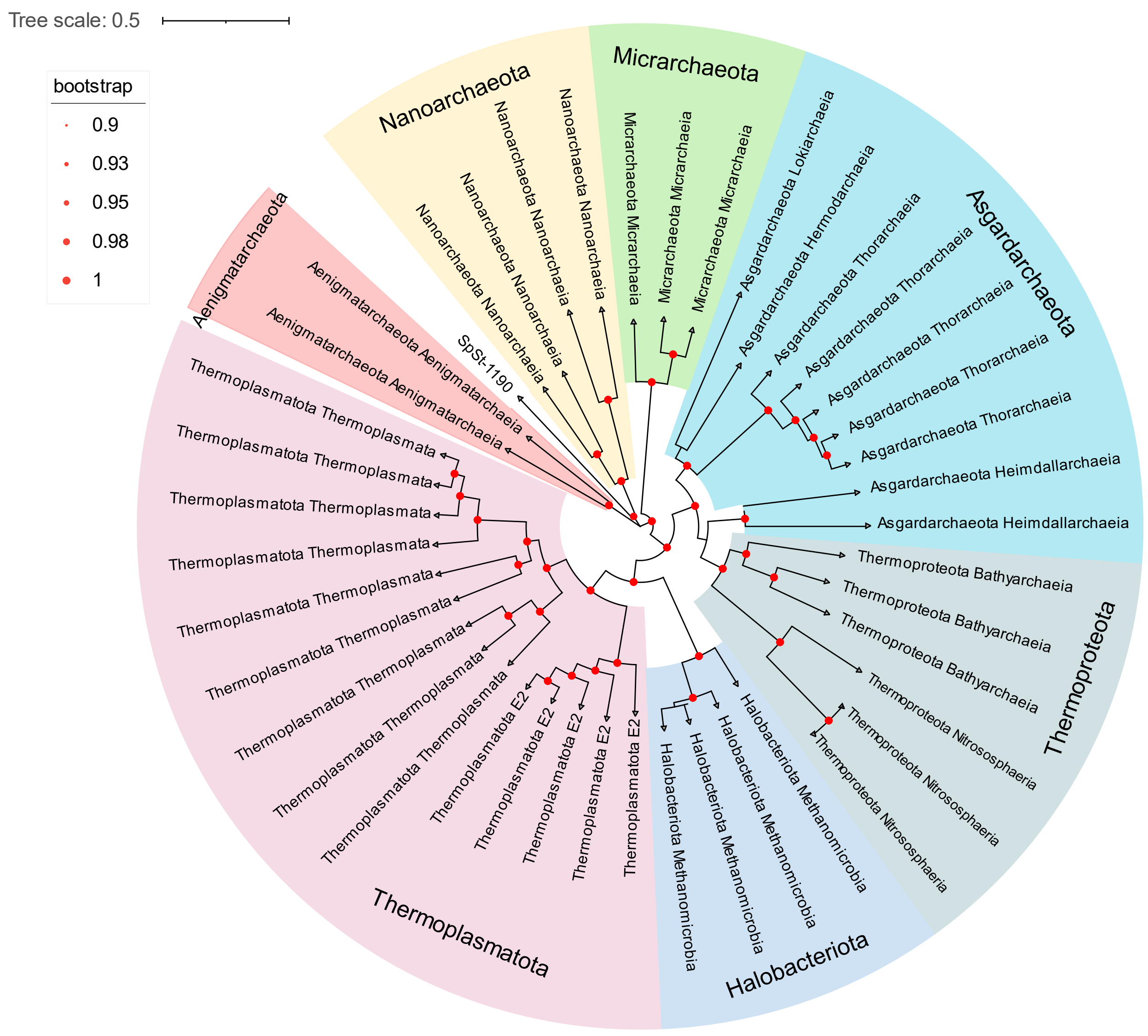

Fig. 5

Figure6
Click here to access/download;Figure;Figures6.pdf

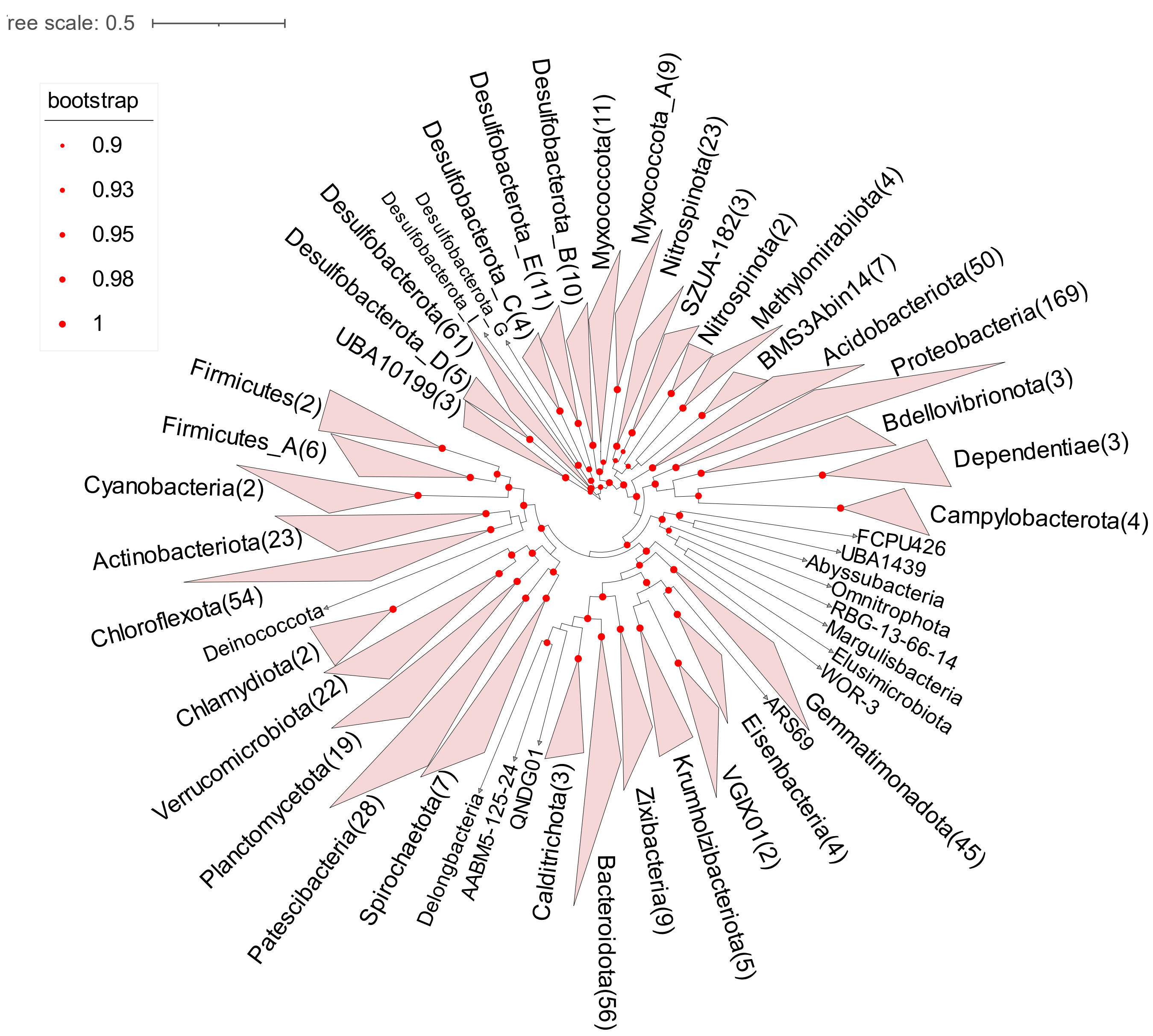

Fig. 6
