# Supplementary material for: A holistic genome dataset of bacteria and archaea of mangrove sediments
Source: Gigascience. 2025 Aug 12;14:giaf081. doi: 10.1093/gigascience/giaf081 (PMC12343073; doi:10.1093/gigascience/giaf081)
Supplement: giaf081_Authors_Response_To_Reviewer_Comments_Original_Submission [file giaf081_authors_response_to_reviewer_comments_original_submission.pdf]

Author's Response To Reviewer Comments

Thank you very much for dealing with our manuscript entitled “A Holistic Genome Dataset of Bacteria and Archaea of Mangrove sediments”. Your comments are constructive and valuable. We have carefully revised our manuscript and addressed all concerns raised by the reviewers. The revised manuscript with highlights on changes and a clean version are provided for your consideration. The point-by-point responses to all the comments are listed as follows:

Reviewer #1 (Comments for the Author):
The authors aimed to investigate A Holistic Genome Dataset of Bacteria and Archaea of Mangrove sediments microbial communities in mangrove sediments across southeast China from 2014 to 2020. combined microbial genomes provide an important complement to global mangrove genome datasets and an understanding of the structure and functions of mangrove microbiomes is not fully convinced. This data is not fully understood by the authors it must be rejected at this stage of the manuscript.

Response: We appreciate the reviewer's concern regarding the depth of analysis in our manuscript. We have substantially enhanced our analysis and discussion of the microbial community structure and functions in mangrove sediments. We have added a comprehensive comparative analysis of microbial community compositions across different geographical locations (lines 171-179), including beta-diversity metrics to quantify community similarities. Furthermore, we performed functional gene profiling using three established databases (MCycDB[1], SCycDB[2], and NCycDB[3]), which allowed use to quantify relative abundances of key functional genes across all samples, as illustrated in Figure 3. We have also expanded our discussion (lines 212-242) to address the potential roles of key bacteria and archaea in biogeochemical cycles, their ecological implications for mangrove wetland ecosystem function.

References:
1. Qian L, Yu X, Zhou J, Gu H, Ding J, Peng Y, et al.. MCycDB: A curated database for comprehensively profiling methane cycling processes of environmental microbiomes. Molecular Ecology Resources. 2022; doi: 10.1111/1755-0998.13589.
2. Yu X, Zhou J, Song W, Xu M, He Q, Peng Y, et al.. SCycDB: A curated functional gene database for metagenomic profiling of sulphur cycling pathways. Molecular Ecology Resources. 2021; doi: 10.1111/1755-0998.13306.
3. Tu Q, Lin L, Cheng L, Deng Y, He Z. NCycDB: a curated integrative database for fast and accurate metagenomic profiling of nitrogen cycling genes. Wren J, editor. Bioinformatics. 2019; doi: 10.1093/bioinformatics/bty741.

No clarity on what basis the author performed the archaeal and bacterial MAGs and MSMs catalogs.

Response: We appreciate the reviewer's question regarding our methodology for constructing the archaeal and bacterial MAGs and MSMs catalogs. Our approach followed a systematic and well-defined workflow. Starting with 82 sediment samples collected from six representative mangrove nature reserves across five provinces in southeastern China, we employed a comprehensive pipeline to generate high-quality MAGs. The process included DNA extraction, high-throughput sequencing, rigorous quality control, metagenomic assembly, and careful binning procedures. From the initial recovery of 1,428 metagenome-assembled genomes (MAGs), we applied stringent quality filters following the Minimum Information about a Metagenome-Assembled Genome (MIMAG) criteria. This resulted in 966 medium- to high-quality MAGs that constitute our final MSM catalog. The specific quality parameters, including completeness (≥50%), contamination (≤10%), strain heterogeneity, and the presence of rRNA and tRNA genes, are detailed in the Methods section (lines 317-325). Each MAG in our catalog meets or exceeds these established community standards, ensuring a robust and reliable genomic resource for mangrove microbiome studies.

The discussion section is one of the most important parts of the paper, this section must be improved with more attention and explanation.

Response: We thank the reviewer for this suggestion. In following your advice, we have enhanced the

discussion section by incorporating comprehensive discussions of microbial community composition and their ecological roles in lines 212-242. Specifically, we now provide:
1. A detailed discussion of the dominance patterns between bacteria and archaea, explaining their differential roles in energy flow and nutrient cycling in mangrove ecosystems. We discuss how our MSMs catalog revealed 58 distinct prokaryotic phyla, with dominant archaeal phyla (Thermoplasmatota, Thermoproteota, Asgardarchaeota) and bacterial phyla (Proteobacteria, Desulfobacterota, Chloroflexota, Acidobacteriota, Gemmatimonadota).
2. An in-depth examination of key functional groups, including sulfate-reducing bacteria (particularly Desulfobacterota), methanogens (8 MAGs from Methylomirabilota and Methanobacteriota), and nitrifying microorganisms (class Nitrososphaeria). We explain their specific roles in biogeochemical cycling and ecosystem functioning.
3. A comprehensive discussion of how these microbial communities contribute to nutrient cycling, organic matter degradation, and pollutant remediation, emphasizing their importance in maintaining ecosystem resilience.

The resolution of all the figures could be higher, and some texts could be easier to see.

Response: We thank the reviewer for this invaluable suggestion, which will enhance the quality of our manuscript. Following up on your suggestion, we have meticulously adjusted the typography and font size of the figures to ensure they are visually more coherent and aesthetically pleasing. Additionally, taking your comments on the resolution and legibility of the texts within the figures to heart, we have not only increased the resolution of all figures but also optimized the text for improved readability.

The manuscript is not well-referenced. All parts of the "Methods section" should be explained in more detail and cited with relevant references However, it would require English editing and checking for typographical errors.

Response: We thank the reviewer for this constructive feedback on the referencing and detail in the “Methods section” of the manuscript. We understand the importance of thorough referencing and clear methodology for the credibility and reproducibility of our research. Addressing your comments, we have carefully reviewed and supplemented the reference information to ensure that each part of the “Methods” section is supported by relevant and up-to-date literature. We have supplemented the reference information and described the processes of sample collection, DNA extraction, sequencing, quality control, splicing, binning and screening with detail, as well as the methods of species annotation, phylogenetic analysis and functional annotation in the methods section. The sampling point information, sampling depth, and sample storage methods have been explicitly mentioned in lines 273-289, providing a clear overview of the experimental setup. In addition, the software used for data processing with detailed parameters have also been added, to ensure transparency and facilitate the replication of the analyses. Besides, we have also conducted a thorough English editing pass and checked for typographical errors to ensure the manuscript is polished and professional. We are grateful for the opportunity to refine our work and hope that these changes meet with your approval.
.
The conclusion is not fully convinced of the real outcome of the manuscript.

Response: We thank the reviewer for the insightful comments regarding the conclusion of the manuscript. We appreciate your feedback and recognize the need to further strengthen the conclusion to ensure consistency with the research findings. Following your guidance, we have taken the following steps to enhance the manuscript:
1. We have enriched the results and discussions by incorporating additional functional analyses of the microbiomes found in mangrove sediments. This additional data provides a more comprehensive understanding of the ecological implications of our findings.
2. The conclusion has been carefully revised to align more closely with the outcomes presented in the manuscript. The revisions are detailed in lines 266-271, where we have articulated the implications of our study more clearly and with greater conviction.
We hope these amendments not only address your concerns but also significantly strengthen the overall narrative and impact of our research.

Reviewer #2 (Comments for the Author):
The author described a holistic genome dataset of bacteria and archaea of mangrove sediments. In general, the manuscript is written well. Kindly refer here for the specific comments.

Abstract:
- The abstract could be improved by providing more information on the prevalence taxa of bacteria and archaea detected in the samples.

Response: We thank the reviewer for this insightful suggestion regarding the improvement of the abstract, which will provide a more comprehensive overview of the key findings from this study. We have expanded the abstract in lines 48-50 to include specific descriptions on the prevalence of the dominant bacterial and archaeal taxa detected in our samples. We have also added results from functional analyses indicating the potential key roles of microbial communities in driving the carbon (C), nitrogen (N), and sulfur (S) cycles within mangrove sediments (in lines 50-52).

Background:
- Line 97: "Error! Reference source not found" appear. Do check for correctness.
Data Description:
- Line 109: "Error! Reference source not found" appear. Do check for correctness.

Response: We thank the reviewer for meticulous review and for bringing these referencing issues to our attention. We appreciate the importance of accurate citations in academic work and grateful for the opportunity to rectify these errors. We have carefully reviewed and corrected the references in the manuscript. The previously mentioned "Error! Reference source not found" issues have been addressed, and the corrected references are now properly cited on lines 99 and 111. We have also taken this opportunity to ensure consistency and accuracy. Additionally, we will implement a more rigorous review process during the manuscript upload to prevent such errors from occurring in the future.

- The methodology and the analysis parts were well written. The use of all relevant software was accurate. The only flaw would be the use of Qubit 2.0, which is a machine to detect DNA quantity, not quality. There should be a step where they use bioanalyzer to determine the library size as well after that.

Response: We appreciate your attention to detail and are grateful for the opportunity to enhance the clarity and rigor of our study. We are very pleased to hear that you found the methodology and analysis parts well written and the use of relevant software accurate. Thank you for your reminder and for correcting the use of Qubit 2.0, we have revised the description of the Qubit 2.0 usage in the “DNA extraction and sequencing” section of the Methods section, as indicated in lines 293-295. Besides, we have added the step to determine the library size using a bioanalyzer, which is now detailed in lines 297-300 of the same section.

Discussion:
- I understand this is a Data Note article type. But I still find the discussion severely lacking in depth. For example, there is no discussion on the distribution of different taxa in different group of samples from different regions. Nor there is any discussion on the role of these bacteria and archaea in the mangrove environment. Which limits the impact and value of the work significantly.

Response: We appreciate the reviewer's constructive feedback regarding the depth of our discussion. We have substantially expanded the discussion to address both the biogeographical distribution patterns and functional aspects of mangrove microbiomes. In lines 171-179, we now provide a detailed comparative analysis of microbial community compositions across different geographical regions, highlighting how environmental factors and local conditions influence community structure. For example, we observed distinct distribution patterns of key phyla (Thermoplasmatota, Thermoproteota, Asgardarchaeota, Proteobacteria, and Desulfobacterota) across our sampling sites, which correlate with regional variations in environmental parameters. Furthermore, we have extensively elaborated on the ecological roles of these microorganisms in mangrove ecosystems (lines 212-242). This includes detailed discussion of their

contributions to: (1) carbon cycling, particularly through the identification of 8 methanogen MAGs from Methylomirabilota and Methanobacteriota; (2) sulfur cycling, emphasizing the role of Desulfobacterota as dominant sulfate-reducing bacteria in anoxic sediments; and (3) nitrogen cycling, notably through the activities of class Nitrososphaeria in nitrification processes. We also discuss how these microbial functions contribute to ecosystem resilience and environmental adaptation in mangrove wetlands.

Editorial Manager®

Cookie Preference Center

We use cookies which are necessary to make our site work. We may also use additional cookies to analyze, improve and personalize our content and your digital experience. For more information, see our Cookie Policy.

You may choose not to allow some types of cookies. However, blocking some types may impact your experience of our site and the services we are able to offer. See the different category headings below to find out more or change your settings.

Allow all

Manage Consent Preferences

Strictly Necessary Cookies

Always active

These cookies are necessary for the website to function and cannot be switched off in our systems. They are usually only set in response to actions made by you which amount to a request for services, such as setting your privacy preferences, logging in or filling in forms. You can set your browser to block or alert you about these cookies, but some parts of the site will not then work.

Functional Cookies

These cookies enable the website to provide enhanced functionality and personalisation. They may be set by us or by third party providers whose services we

have added to our pages. If you do not allow these cookies then some or all of these services may not function properly.

Performance Cookies

These cookies allow us to count visits and traffic sources so we can measure and improve the performance of our site. They help us to know which pages are the most and least popular and see how visitors move around the site.

Targeting Cookies

These cookies may be set through our site by our advertising partners. They may be used by those companies to build a profile of your interests and show you relevant adverts on other sites. If you do not allow these cookies, you will experience less targeted advertising.

Cookie List

Clear

Apply Cancel

Consent Leg.Interest

Confirm my choices

Powered by onetrust
