# Supplementary material for: A holistic genome dataset of bacteria and archaea of mangrove sediments
Source: Gigascience. 2025 Aug 12;14:giaf081. doi: 10.1093/gigascience/giaf081 (PMC12343073; doi:10.1093/gigascience/giaf081)
Supplement: giaf081_Authors_Response_To_Reviewer_Comments_Revision_1 [file giaf081_authors_response_to_reviewer_comments_revision_1.pdf]

Author's Response To Reviewer Comments

Dear Dr Zhang,

We appreciate the opportunity to revise our manuscript titled "A Holistic Genome Dataset of Bacteria and Archaea of Mangrove Sediments" for publication in Giga Science (GIGA-D-24-00229R1) and are grateful for the positive and constructive comments provided by the reviewers. We have studied reviewer's comments carefully and have made revision which marked in blue in the paper. We have tried our best to revise our manuscript according to the comments. Attached please find the revised version, which we would like to submit for your kind consideration.

We would like to express our great appreciation again to you and reviewers your consideration of publishing our manuscript in this journal and for the comments on our paper. We are looking forward to hearing from you.

Yours sincerely,

Yang Liu
Leading contacts: Yang Liu & Meng Li
Institute for Advanced Study, Shenzhen University
Nanhai Ave 3688, Shenzhen, China, 518060
Email: yangliu@szu.edu.cn; limeng848@szu.edu.cn

Response to Reviewers' Comments：
Thank you very much for dealing with our manuscript entitled "A Holistic Genome Dataset of Bacteria and Archaea of Mangrove Sediments". Your comments are constructive and valuable. We have further revised our manuscript to make it more rigorous through systematic language revisions, transparent elaboration of research limitations, and prospects for future research directions. The revised manuscript with highlights on changes and a clean version are provided for your consideration. The point-by-point responses to all the comments are listed as follows:

Reviewer #1 (Comments for the Author):
The Manuscript has not fully convinced by the authors. So I regrets to inform you that, I can't able to review this MS again. It must be rejected based on wrong interpretation on the sequence data.

Response: We appreciate the reviewer's time and critical assessment of our manuscript. We acknowledge the concern regarding potential misinterpretations in the sequence data analysis and take this feedback with utmost seriousness. To address this, we have taken the following actions in the revised manuscript:

1. We rigorously revised the language in the manuscript, adjusted the discussion and conclusion of possible over-interpretation of data, and avoided absolute expressions that might imply causality. Examples include:
Abstract: We adjusted the sentence from "indicate the potential roles of microbial communities in helping to drive the carbon, nitrogen and sulfur cycling" to "suggest that the microbial communities may contribute to the carbon, nitrogen and sulfur cycling" (lines 50-52). Also, we adjusted the sentence from "should greatly facilitate our understanding of the compositions and functions of mangrove sediment microbiomes" to "may serve as a foundational resource for enhancing our understanding of the compositions and functions of mangrove sediment microbiomes" (lines 52-55).
Background: We adjusted "the data set of mangrove sediment microbiomes will be a very valuable resource, which will help us enhance the understanding of the composition, structure and function of mangrove sediment microbiomes" to "the dataset of mangrove sediment microbiomes represents a valuable resource, which could enhance our understanding of the composition, structure and putative functional roles of mangrove sediment microbiomes" (lines 76-78).
Data description: We clarified gene abundance trends as "relative differences" rather than definitive functional evidence (lines 151-153).

Discussion: We incorporated cautious modifiers (e.g., "may", "likely", "potentially") throughout the manuscript to frame hypotheses about microbial ecological roles, thereby avoiding overstatement of causal relationships. For example, assertions about nutrient cycling were revised from definitive claims (e.g., "influences", "the distribution of") to probabilistic language (e.g., "may influences", "be correlated with") in lines 192-194. In lines 214-215, we adjusted "one of the main drivers of the element cycling in mangrove sediments" to "likely play a significant role in element cycling in mangrove sediments". In lines 227-230, the phrase "drove the selection of" was revised to "are associated with" to reflect the correlative nature of metagenomic inferences. In lines 256-258, the phrase "will be replaced" was revised to "could potentially be supplemented" to more accurately reflect the provisional nature of MAGs (Metagenome-Assembled Genomes) while acknowledging that future advancements may enhance, rather than entirely replace these genomes.

2. We added an appropriate affirmation to the limitations of the dataset in our discussion and conclusion (lines 239-249 and 277-294), detailing the following limitations. To begin with, the mean completeness is 75.6% and the mean contamination rate is 4.1%, which may affect the accuracy of functional predictions, especially inference of low-abundance taxa or novel pathways (e.g., unannotated genes in the "Function unknown" category). Additionally, we acknowledged that the functional annotation relies on homology databases (e.g., KEGG, eggnog 5.0), which are inherently limited in annotating uncharacterized genes. Finally, we clarified that sampling focused on six reserves in southeastern China, limiting extrapolation to mangrove ecosystems with distinct environmental conditions (e.g., hypersaline estuaries or heavily polluted coastal zones).
Despite the limitations inherent to metagenome-assembled genomes (MAGs), this study provided three key contributions to mangrove microbiome research. First of all, it established a foundational MAG database spanning 58 prokaryotic phyla, significantly expanding the genomic representation of mangrove ecosystems globally. Secondly, it generated hypothesis-driven insights into critical biogeochemical processes, such as methane cycling (mcrA/pmoB genes) and sulfate reduction (dsrAB in Desulfobacterota), which prioritize targets for experimental validation via metatranscriptomics or stable isotope probing. While these MAGs are provisional placeholders, they may catalyze future work to resolve mangrove-microbe interactions through expanded sampling, multi-omics integration, and targeted cultivation of functionally critical taxa.

3. To validate ecological correlations among mangrove wetland microorganisms in future research, we proposed future validation directions to confirm ecological correlations among mangrove wetland microorganisms. Specifically, future studies should systematically integrate cultivation-based approaches, multi-omics analyses, and in situ measurement techniques to experimentally verify microbial metabolic pathways. (lines 249-253)

We are grateful for the opportunity to refine our work and hope that these changes meet with your approval. We welcome further guidance if specific analyses remain unclear.

Reviewer #2 (Comments for the Author):
The authors have done a good job in revising the work according to my comments. I hereby recommend the acceptance of the writing.

Response: Thank you for your positive feedback and constructive comments on our manuscript. We greatly appreciate your recognition of our revisions and your recommendation for acceptance. It is encouraging to know that our efforts to address the concerns raised in the review process have met your approval.

Editorial Manager®

Cookie Preference Center

We use cookies which are necessary to make our site work. We may also use additional cookies to analyze, improve and personalize our content and your digital experience. For more information, see our Cookie Policy.

You may choose not to allow some types of cookies. However, blocking some types may impact your experience of our site and the services we are able to offer. See the different category headings below to find out more or change your settings.

Allow all

Manage Consent Preferences

Strictly Necessary Cookies

Always active

These cookies are necessary for the website to function and cannot be switched off in our systems. They are usually only set in response to actions made by you which amount to a request for services, such as setting your privacy preferences, logging in or filling in forms. You can set your browser to block or alert you about these cookies, but some parts of the site will not then work.

Functional Cookies

These cookies enable the website to provide enhanced functionality and personalisation. They may be set by us or by third party providers whose services we have added to our pages. If you do not allow these cookies then some or all of these services may not function properly.

Performance Cookies

These cookies allow us to count visits and traffic sources so we can measure and improve the performance of our site. They help us to know which pages are the most and least popular and see how visitors move around the site.

Targeting Cookies

These cookies may be set through our site by our advertising partners. They may be used by those companies to build a profile of your interests and show you relevant adverts on other sites. If you do not allow these cookies, you will experience less targeted advertising.

Cookie List

Clear

Apply Cancel
Consent Leg.Interest

Confirm my choices

Powered by onetrus
